# RNA-viromics reveals diverse communities of soil RNA viruses with the potential to affect grassland ecosystems across multiple trophic levels

**DOI:** 10.1038/s43705-022-00110-x

**Published:** 2022-04-08

**Authors:** Luke S. Hillary, Evelien M. Adriaenssens, David L. Jones, James E. McDonald

**Affiliations:** 1 School of Natural Sciences, Bangor University, Bangor, Gwynedd LL57 2UW UK; 2 Quadram Institute Bioscience, Norwich Research Park, Norwich, NR4 7UQ UK; 3 SoilsWest, Centre for Sustainable Farming Systems, Food Futures Institute, Murdoch University, Murdoch, WA 6105 Australia

**Keywords:** Microbial ecology, Metagenomics, Soil microbiology

## Abstract

The distribution and diversity of RNA viruses in soil ecosystems are largely unknown, despite their significant impact on public health, ecosystem functions, and food security. Here, we characterise soil RNA viral communities along an altitudinal productivity gradient of peat, managed grassland and coastal soils. We identified 3462 viral contigs in RNA viromes from purified virus-like-particles in five soil-types and assessed their spatial distribution, phylogenetic diversity and potential host ranges. Soil types exhibited minimal similarity in viral community composition, but with >10-fold more viral contigs shared between managed grassland soils when compared with peat or coastal soils. Phylogenetic analyses predicted soil RNA viral communities are formed from viruses of bacteria, plants, fungi, vertebrates and invertebrates, with only 12% of viral contigs belonging to the bacteria-infecting *Leviviricetes* class. 11% of viral contigs were found to be most closely related to members of the *Ourmiavirus* genus, suggesting that members of this clade of plant viruses may be far more widely distributed and diverse than previously thought. These results contrast with soil DNA viromes which are typically dominated by bacteriophages. RNA viral communities, therefore, have the potential to exert influence on inter-kingdom interactions across terrestrial biomes.

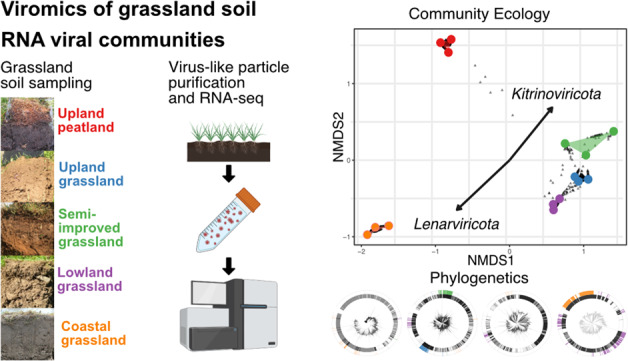

## Introduction

Viruses are the most common and diverse biological entities on Earth [1] and can exert significant influence on their hosts. In addition to their ecological functions, viruses are key influencers of public health and food security, causing 47% and 44% of plant and human emerging infectious diseases, respectively [2, 3]. The current COVID-19 pandemic highlights the critical importance of understanding the role of viruses in the environment, and how natural and anthropogenic ecosystems can function as sources of novel zoonotic infections. Grassland ecosystems form 30–40% [4] of total land cover and provide essential ecosystem services, including food production, flood mitigation and carbon storage [4, 5]. Within these, and other terrestrial ecosystems, DNA viruses are known to play essential roles in microbial community dynamics and carbon biogeochemical cycling [6–10], yet the role of viruses within these critical ecosystems remains undercharacterised [11] and in particular, our knowledge of soil RNA viruses is significantly limited [12, 13]. To date, soil viral ecology has focused almost exclusively on DNA viruses of bacteria and archaea. In contrast, marine DNA and RNA viruses have been characterised on an ocean-wide scale [14], and the significant level of diversity observed suggests that the global virome could be the largest reservoir of genetic diversity on the planet [15].

The vast majority of known RNA viruses lie within the realm *Riboviria* and possess a universally conserved RNA-dependent RNA polymerase (RdRP) gene [16]. This gene can be used to identify viral RNA genomes and genome fragments from large-scale metatranscriptome datasets. A number of recent studies have used this strategy to dramatically increase the number of known RNA viral sequences [12, 17, 18] allowing the construction of a broad global viral taxonomy [19]. Difficulties in generating and analysing environmental RNA viral sequencing data remain, largely due to experimental challenges of extracting sufficient viral RNA from environmental samples, and in computationally identifying RNA viral genome fragments in large metatranscriptome datasets [20].

The detection of viral genome fragments in soils can be enhanced by enriching, concentrating and purifying virus like particles (VLPs) from the soil matrix. Viromics uses size selection to enrich for VLPs in environmental samples, ensuring that they represent a greater proportion of the data obtained from high throughput sequencing [21]. This can significantly improve the quality and quantity of viral genomes recovered from soils over bulk-soil metagenomes and metatranscriptomes [22]. Viral RNA for use in viromics studies can be readily extracted from water, sewage and sediments [23–25] and it is possible to detect RNA viral sequences in bulk soil and rhizosphere metatranscriptomes [12, 13] but to date, and to the best of our knowledge, there has been no published attempt to apply viromics to the study of RNA viruses in soil.

Here, we use viromics to characterise the soil RNA viral communities of five contrasting soil types along a typical temperate oceanic grassland altitudinal productivity gradient [26]. We identified RdRP-containing viral contigs and examined their distribution across different soil types at both a viral contig and phylum level. We then used phylogenetic analyses to place these viral contigs within phylogenetic trees of known viruses, and compared them to viral contigs detected by a previous mesocosm bulk soil meta-transcriptomics study [12]. Our findings demonstrate that soils represent a significant reservoir of viral diversity that have the potential to impact not just the soil microbial community, but also across multi-kingdom host ranges.

## Materials and methods

### Field site description, soil sampling and processing

Five sites along an altitudinal gradient at Henfaes Research Centre, Abergyngregyn, Wales were sampled on 31st October 2018. Three adjacent 5 × 5 m plots were marked out at each site and ~2 kg of soil was extracted from each site between 0 and 10 cm depth using a 3 cm diameter screw auger with evenly spaced sampling within each grid. The augur was cleaned with disinfectant and a dummy soil core taken and discarded outside of the sampling area prior to sampling each plot. Soil from each plot was sieved separately to 2 mm and stored in 100 g aliquots at −80 °C prior to RNA extraction.

### Viral RNA enrichment and extraction

Virus-like particle extraction was based on protocols developed by Trubl et al. [27] and Adriaenssens et al. [23]. A total of 16 samples, three per site and one 100 mL PCR-grade water negative extraction control were processed separately. 100 g of soil per sample was thawed and evenly divided into eight 50 mL centrifuge tubes (12.5 g of soil per tube, hereon referred to as subsamples). Each subsample was suspended in 37.5 mL of amended potassium citrate buffer (1% potassium citrate, 10% phosphate-buffered saline, 5 mM ethylenediaminetetraacetic acid (EDTA), and 150 mM magnesium sulphate (MgSO_4_), 300 mL total volume per sample). Each subsample was subjected to 30 s manual shaking followed by 60 s vortexing at maximum speed. After physical disruption, subsamples were placed on ice on an orbital shaker and shaken at 300 rpm for 30 min and then centrifuged for 30 min at 3000 × g, 4 °C. Supernatants were removed to new centrifuge tubes and polyethylene glycol, (PEG - 6000 MW) and sodium chloride (NaCl) were added to 15% (w/v) and 2% (w/v) respectively to precipitate VLPs overnight at 4 °C. Precipitates were recovered by centrifuging tubes for 80 min at 2500 × *g*, 4 °C and discarding the supernatants. The eight subsample pellets from each 100 g soil sample were recombined by resuspending them in a total volume of 10 mL of Tris buffer (10 mM Tris-HCl, 10 mM MgSO_4_, 150 mM NaCl, pH 7.5). Recombined samples were then filtered through sterile polyethersulfone (PES) 0.22 μm pore size syringe filters and concentrated to <600 μL using Amicon Ultra-15 centrifugal filter units (50 kDa MWCO, Merck) prior to RNA extraction.

All RNA extraction protocols were used according to the manufacturer’s instructions except where specified. Nucleic acids were extracted using the AllPrep PowerViral DNA/RNA extraction kit (Qiagen) with the addition of 10 µL/mL 2-β-mercaptoethanol. Co-purified DNA was DNase digested using the Turbo DNA Free kit (Thermo Fisher) using two sequential 30-min incubations at 37 °C, each using 1 U of Turbo DNase. DNase was inactivated and removed using the supplied DNase inactivation resin and RNA was further purified using the RNA min-elute kit (Qiagen). Unlike the DNase equivalent commonly used in DNA virome protocols, no pre-extraction RNase treatment was performed as this has previously been suggested to be detrimental to RNA viral recovery [23].

### Library preparation, sequencing and initial short read QC

Sequencing libraries were prepared using total RNA without mRNA isolation or rRNA depletion using the NEBNext Ultra Directional RNA Library Prep Kit (New England Biolabs) by the Centre for Genomics Research (CGR), University of Liverpool. Initial fragmentation, denaturation and priming for cDNA synthesis were performed with an incubation time of 7 min at 94 °C and random primers. In total, 17 libraries were prepared using unique dual indexes and pooled: 15 soil virome samples from five sites, plus one extraction negative control and one library construction negative control of PCR-grade water which were processed alongside the samples and during sequencing library production. A volume of each negative control library equal to the largest volume from a soil virome sample was added to the final pool. Libraries were pooled and sequenced (150 bp paired end) on one lane of a HiSeq 4000.

### RNA virome data analysis

Initial demultiplexing and quality control performed by CGR removed Illumina adapters using Cutadapt version 1.2.1 [28] with option -O 3 and Sickle version 1.200 [29] with a minimum quality score of 20. Libraries were further filtered by removing reads with a read length <35 bp, a GC percentage of <5% or >95%, or a mean quality score <25, using Prinseq-lite v0.20.4 [30]. Ribosomal reads were removed using SortMeRNA v3.0.3 [31] using default parameters. Reads from each library were pooled, error-corrected using tadpole.sh (mode = correct ecc=t prefilter=2) and deduplicated with clumpify.sh (dedupe subs=0 passes=2) from the BBTools package (v37.76: sourceforge.net/projects/bbmap/). Reads from all libraries were co-assembled using MEGAHIT 1.1.3 [32] (–k-min 27, –k-max 127, –k-step 10, —min-count 1).

### Identification and abundance of viral sequences

Assembled contigs were compared to the NCBI nr complete database (downloaded on 27th November 2019) using Diamond BLASTx [33] (—sensitive, —max-target-seqs 15, —evalue 0.00001) and taxonomic assignments made using MEGAN v6 [34]. All contigs with hits matching cellular organisms or dsDNA/ ssDNA viruses were excluded from subsequent analysis.

HMMs used in RdRP detection were generated from alignments previously published by Wolf et al. [16]. Protein coding genes in contigs >300 bp in length were predicted using Prodigal v2.6.3 (-p meta) [35] and searched for RdRP genes using HMMSearch [36]. Contigs with *E* values < 0.001 and scores >50 were clustered with CD-Hit v4.8.1 [37] to 95% average nucleotide identity across 85% alignment fraction [38]. Each contig was assigned a broad taxonomic classification based on HMMsearch results. Contigs with hits from more than one RdRP phylum were assigned to the classification with the lowest E-value.

Reads were mapped to contigs using BBwrap (vslow = t minid=0.9 - https://sourceforge.net/projects/bbmap/) and contigs with any mapped reads from either the negative extraction control or the negative library-preparation control were excluded from further analysis. Viral contigs with a horizontal genome coverage of >50% were determined as present for each sample. Any viral contig with coverage of <50% had its abundance reset to zero. Fragments per kilobase million values calculated by BBwrap were converted to Counts Per Million (CPM) using the fpkm2tpm function from the R package RNAontheBENCH [39].

### Ecological data analysis

Community analysis was performed using R and the Vegan package [40]. UpSet plots were produced to highlight contigs shared between sampling sites based on the combined collection of viral contigs identified as present at each site. Separate UpSet plots for contigs shared between sampling replicates are contained within Supplementary Fig. 5. A table of CPM values for each viral contig in each sampling replicate for each site was used to calculate α-diversity metrics (richness, Simpson and Shannon diversity indexes).

The same table was used to generate a β-diversity distance matrix (Bray-Curtiss) using the function metaMDS within the Vegan package. Statistically significant differences between sampling sites were tested for using Kruskall-Wallis (α-diversity metrics) and PERMANOVA (β-diversity). Co-ordinates for individual viral contigs were taken from expanded scores based on a Wisconsin (square root) transformation of the CPM value matrix. Summed CPM values for each phylum were fitted to the NMDS ordination plot using the function env_fit and phyla with a *p* value of <0.05 displayed as vectors. Data visualisation was performed using the packages ggplot2 and upsetR [41, 42].

### Phylogenetic analysis

RdRP sequences from contigs produced by Starr et al. [12] were identified and processed by the same methods described above. These were pooled with those identified by this study and those identified by Wolf *et al*. [16]. Sequences were then aligned using MAFFT v7.427 [43] (—retree 2 —maxiterate 2) and trees generated with FastTree v2.1.11 [44] (-wag -spr 4 -mlacc 2 -pseudo -slownni). Trees were visualised with iToL [45] and annotated with the aid of table2itol (https://github.com/mgoeker/table2itol).

## Results and discussion

### Viromics reveals extensive diversity in soil RNA viral communities

**Fig. 1:**
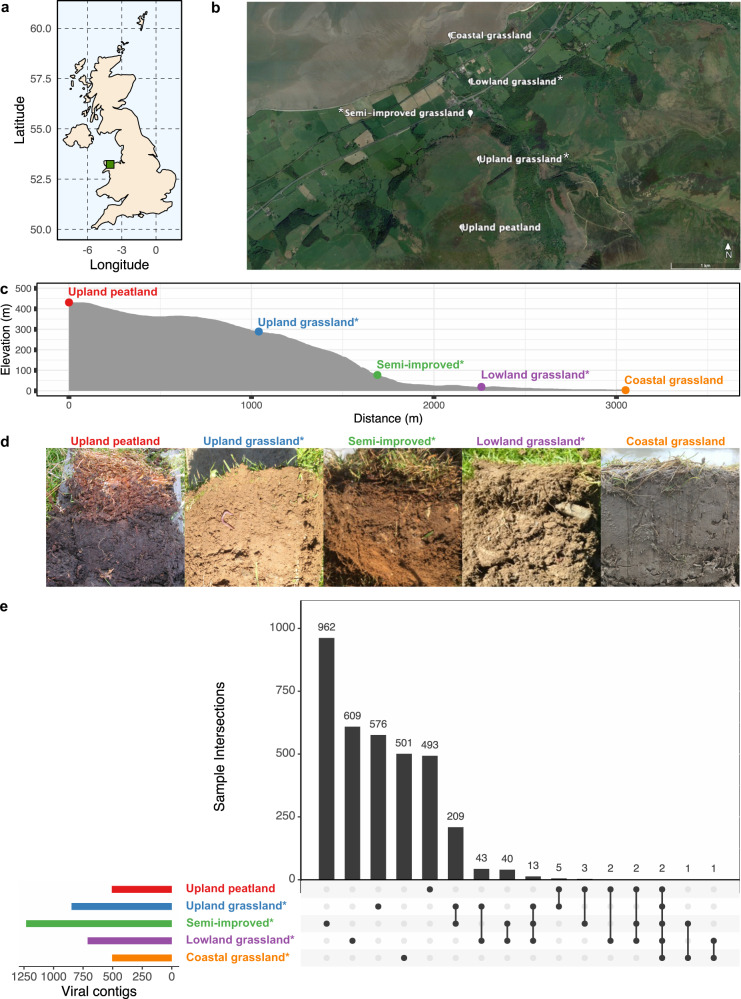
Soil samples were taken along an altitudinal primary productivity gradient in North Wales, UK (**a**). Sampling sites included upland peatland, (*) three forms of grassland under different management regimes (unimproved upland, semi-improved and improved lowland grassland) and unmanaged coastal grassland (**b** - made using Google Earth Pro). Elevation varied by 400 m along the transect (**c**). Site co-ordinates and soil descriptions can be found in Supplementary Table 1. Images of soils from each site can be seen in (**d**) from left to right: upland peatland, upland grassland, semi-improved grassland, lowland grassland and coastal grassland. An Upset plot of the distribution of identified viral contigs (**e**) demonstrates how whilst the majority of viral contigs are found solely at each site, the managed grassland sites share more viral contigs in common than with the upland peat or coastal grassland sites, with the coastal grassland site being almost completely unique.

In this work, we characterised the soil RNA viromes of five contrasting soil types along a typical temperate oceanic grassland altitudinal productivity gradient (Fig. 1a–d, further described in Supplementary Table 1) [26]. Raw reads were filtered for quality and rRNA contamination. The percentage of rRNA reads in each library was highly variable (0.5–95% of total reads) but did not impact the amount or percentage of reads mapping to the collection of assembled viral contigs (Spearman rank correlation, *p* = 0.667 and *p* = 0.611 respectively, (see Materials and Methods section, summary statistics on rRNA read removal and read mapping are provided in Supplementary Fig. 1). Future viromics work would benefit from rRNA removal, assuming the yield of purified viral RNA is sufficient for sequencing library construction. Pre-viral lysis RNase digestion has been used previously to achieve this but this can also remove substantial quantities of viral RNA as well [23]. Filtered sequencing reads from all libraries were co-assembled and contigs >300 bp were used in further analysis. Genes were predicted by Prodigal [35] and searched for the RNA viral hallmark gene RNA dependent RNA polymerase (RdRP) using HMMER [36] and five Hidden Markov Models (HMMs) built from multiple sequence alignments of the RdRPs from the five major *Riboviria* viral phyla [16].

A total of 3471 contigs containing putative viral RdRP genes were taken forward for further analysis. 16 additional contigs were excluded where ≥1 read mapped to the contig from either the negative extraction, or negative library preparation control libraries, of which one had a horizontal coverage of >50%, but only in the negative extraction control. Although a clustering step on the co-assembled contigs was performed at 95% identity over 85% of the contig length, matching the thresholds established for demarking dsDNA viral species [38], all clusters from this dataset contained a single viral contig. As boundaries for RNA viral operational taxonomic units are yet to be established, the term “viral contig” has been used in place of vOTU for the purposes of this study. Read mapping was used to identify 3462 viral contigs present within a sample where the horizontal genome coverage was ≥50%. Read mapping and contig coverage statistics are provided in Supplementary Figs. 1–4.

An UpSet plot of viral contigs shared between sites (Fig. 1e) shows that few were common between sites (0.79–32% per site) with the managed grassland sites showing the most similarity. 97–99% of viral contigs shared by managed grassland sites were shared with at least one other managed grassland site (see Supplementary Fig. 5 for the distribution of contigs shared between replicates of each site). The coastal grassland site shared the least viral contigs with any other site (4 in total), whilst the upland peatland site, although markedly different, shared more viral contigs in common with managed grassland sites it was geographically closer to (7 with the upland-grassland site, 14 overall). This could reflect similarities between those habitats, or result from viral particles being transferred between these habitats by ground/surface water runoff. As the horizontal coverage threshold used to determine viral contig presence can influence the sensitivity and precision of detection [46], the same analysis was repeated with 25%, 75% and 95% horizontal genome coverage and the same pattern of higher overlap between managed grassland sites than with upland peatland and unmanaged coastal grassland sites was repeated (see Supplementary Fig. 2).

**Fig. 2:**
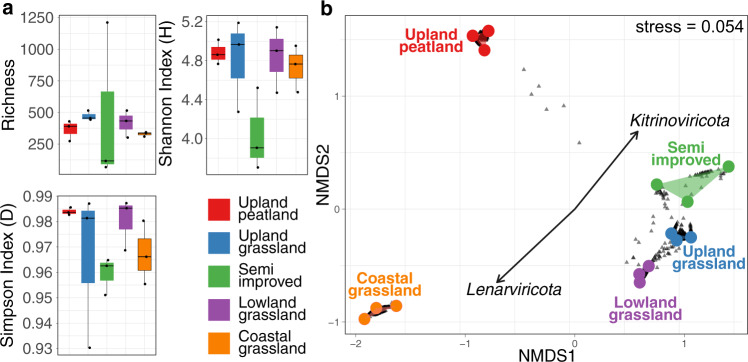
(**a**) α-diversity metrics and (**b**) β-diversity NMDS ordination of viral contig relative abundance in five contrasting soil types along an altitudinal primary productivity gradient. No statistically significant differences between sites were found in richness (Kruskall-Wallis, *p* = 0.369), Simpson index (Kruskall-Wallis, *p* = 0.138) or Shannon index (Kruskall-Wallis, *p* = 0.264) but a significant difference in β-diversity (Bray-Curtis) was found (PERMANOVA, *R*^2^ = 0.738, *P* < 10^−4^). Managed grassland sites cluster closely together in the NMDS ordination whilst peatland and coastal RNA viromes are clearly separated. Whilst a small number of viral contigs (grey triangles) can be seen to be shared between upland-peat and grassland sites, the coastal grassland site is distinctly separate. Phylum-level CPM values were fitted to the ordination plot to determine which phyla were driving community difference, with *Lenarviricota and Kitrinoviricota* showing significant effects (*p* < 0.05).

Relative abundance was calculated using mapped reads normalised by contig length and library size (CPM—counts per million, see Materials and Methods) for viral contigs identified as present in each sample. No significant differences in α-diversity were found between the five sites, with Simpson diversity index ranging between 0.93 and 0.99, indicating that all sites are highly diverse (Fig. 2a). Although no overall difference in richness was observed, the semi-improved grassland site showed substantially higher range in viral contig relative abundance than the other four sites, possibly due to increased site heterogeneity.

β-diversity was analysed via non-metric multidimensional scaling (NMDS—Fig. 2b) and phylum-level CPM values fitted to the ordination plot. Each site is separate and significantly distinct (PERMANOVA, *R*^2^ = 0.738, *P* < 10^−4^). Dense collections of viral contigs (grey triangles) are located between the sampling replicates of each site, with samples from managed grassland sites positioning closer together than to samples from upland peatland or unmanaged coastal grassland sites. Figures 1e and 2b also show the lack of a clear core soil RNA virome at the viral contig level, and that a combination of soil type, plant coverage and land management may be determining factors of soil RNA viral abundance and diversity.

### Habitat affects phylum level RNA viral community structure

**Fig. 3:**
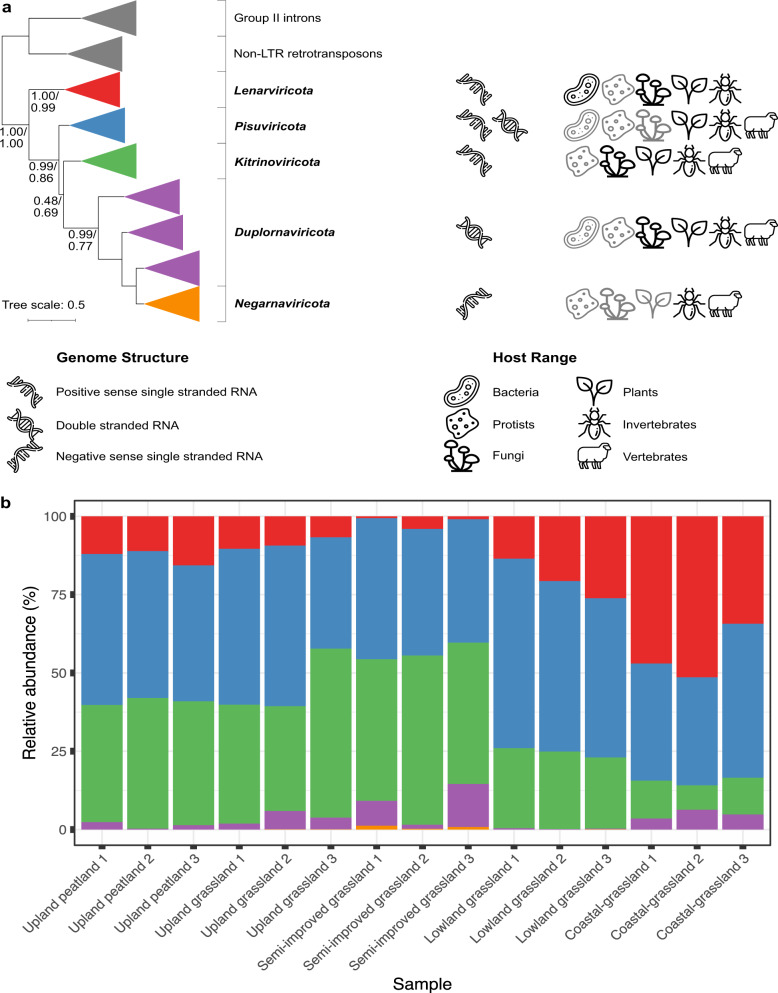
Phylogenetic organisation, genome structure and host range (**a**) of the five phyla of RNA viruses within the *Riboviria* realm, based on RdRP multiple sequence alignments (adapted from Wolf et al. [16]). Grey host icons indicate limited known numbers of viruses infecting these hosts. Viral contigs from three independent samples of five contrasting soil types along an altitudinal primary productivity gradient were classified into one of these five phyla and relative abundances calculated from CPM values of mapped reads (**b**). *Pisuviricota* remain broadly similar between samples whilst *Lenarviricota* and *Kitrinoviricota* increase and decrease in proportion respectively, moving from upland to coastal sampling sites.

To explore broader similarities shared between sites, contigs containing RdRP genes were classified based on the broad phylogenetic scheme constructed by Wolf et al. [16]. This divides the *Riboviria* realm into five phyla, based on RdRP amino acid multiple sequence alignments: positive-sense single-stranded *Lenarviricota, Pisuviricota* (including some double-stranded RNA viruses) and *Kitronoviricota*, double-stranded *Duplornaviricota* and negative-sense single-stranded *Negarnaviricota*, which form a clade located within the *Duplornaviricota* (see Fig. 3a) [16, 19]. The proportion of *Lenarviricota* members increases, whilst the proportion of contigs assigned to the phylum *Kitrinoviricota* decreases when comparing lowland and upland sites (Fig. 3b) and these two phyla are significant drivers of differences in β-diversity as indicated in Fig. 2b. As the lowland sites are also closer to the coastline (Fig. 1b) and other soil characteristics co-vary with altitude, it is difficult to identify the environmental drivers of this difference. In contrast, the relative abundance of *Pisuviricota* members stays broadly similar between each site.

The RNA viromes are all heavily dominated by positive-sense single-stranded RNA (+ssRNA) viruses, with the double-stranded RNA (dsRNA) *Duplornaviricota* far fewer in relative abundance and mostly observed in the semi-improved and coastal grassland samples. Only four negative-sense *Negarnaviricota* RNA (-ssRNA) viral contigs were identified in the whole study and these were exclusively found in the managed grassland sites.

### Phylogenetic analyses reveal expanded fine-scale RNA viral diversity

**Fig. 4:**
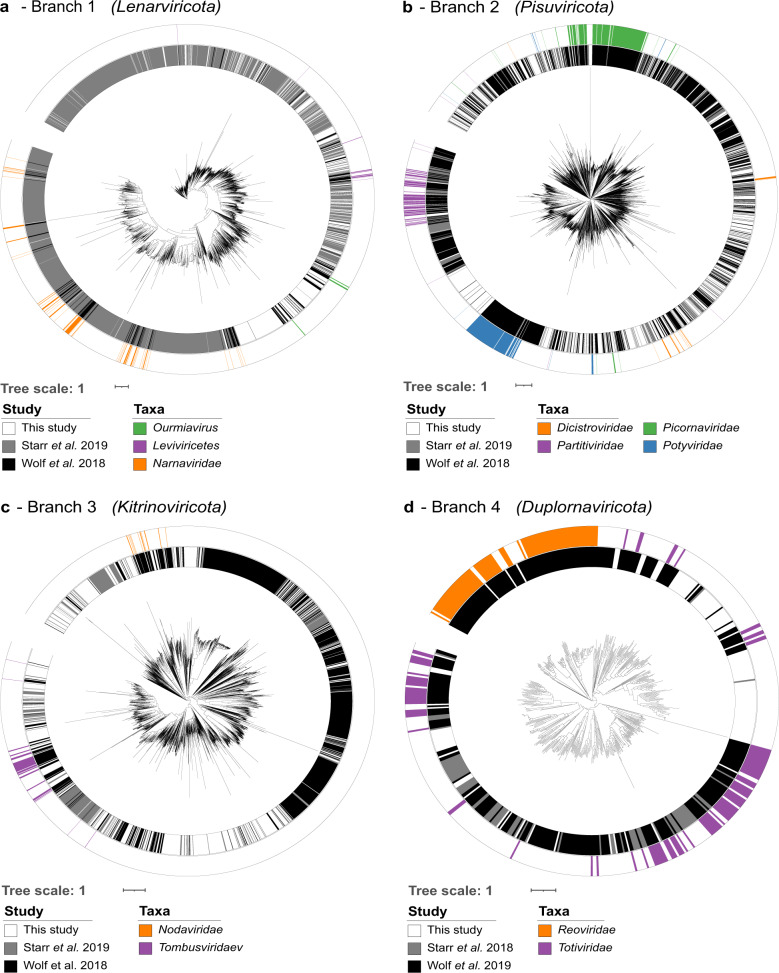
Phylogenetic trees of RdRP genes based on protein multiple sequence alignments. Sequences found across all soil sites from this study (inner ring, white) were aligned with those used to construct the RNA global taxonomy (Wolf *et al*. inner ring, black) and another soil study (Starr *et al*. inner ring, grey). Global RNA phylogeny is divided into the five proposed RNA viral phyla (**a**) *Lenarviricota*, (**b**) *Pisuviricota* (the picornavirus supergroup), (**c**) *Kitrinoviricota*, (**d**) *Duplornaviricota*, and *Negarnavirota* (not featured). RdRP genes with established phylogeny of interest are highlighted separately in each panel.

To explore the phylogeny of the viruses discovered in this study further, protein alignments of RdRP genes for viruses in this study, reference viruses from Wolf et al. [16] and sequences from a recently published bulk soil and leaf litter metatranscriptomics study by Starr et al. [12] were used to generate phylogenetic trees (Fig. 4, more detailed trees are found in Supplementary Fig. 6). Many viruses found in this study appear as blocks of closely related viruses containing few reference sequences, similar to the observations of Starr et al. [12] In other regions of the phylogenetic trees, e.g. *Pisuviricota and Kitrinoviricota* (Fig. 4b and c), novel viruses are fewer in number and evenly distributed across the known RdRP phylogeny.

The phylogenetic tree for the phylum *Lenarviricota* (Fig. 4a) can be divided into three sections containing reference viruses belonging to the classes *Leviviricetes*, the family *Narnaviridae* and related mitoviruses, and the genus *Ourmiavirus*, found within the newly reclassified family *Botourmiaviridae* [19, 47]. Similarly to the work of Starr et al. [12], this study has detected a large number of potential leviviruses (416 in total, see Fig. 4a, outer, purple, top right quadrant and Supplementary Fig. 7). Isolated members of the class *Leviviricetes* predominantly infect proteobacteria and their known diversity has recently been significantly expanded [18, 48]. We found comparatively few *Narnaviridae (21 in total)* and this may be linked to their structure: *Narnaviridae* members are capsidless +ssRNA viruses that encode no structural proteins and are obligately intracellular [49]. As viromics approaches isolate intact virions, narnaviruses would most likely not be enriched using this technique. No pre-lysis RNase digestion was performed in this study and those few examples detected here may have been released into the environment from soil processing causing damage to host cells. In contrast, the encapsidated genus *Ourmiavirus*, within the family *Botourmiaviridae*, comprises plant pathogens with segmented genomes of three ssRNA molecules, each carrying genes for a RdRP, movement protein or capsid protein. 377 ourmia-like viral contigs were identified and are almost exclusively found in managed grassland or upland peat sites (Supplementary fig. 7), suggesting that this genus of plant viruses may form a larger, more diverse and undercharacterised clade of grassland plant viruses within the *Botourmiaviridae* family. The recovery of complete segmented viral genomes from metagenomics or viromics datasets is particularly challenging due to their segmented nature. Previous studies have used co-occurrence of viral contigs in other publicly available datasets [50] or sequence homology to known viral species [51], however the lack of suitable publicly available datasets and extensive horizontal gene transfer can hamper these efforts. Unlike other members of the *Botourmiaviridae* family, viruses of the genus *Ourmiavirus* are known to possess coat proteins that show similarity with highly disparate viruses spanning multiple phyla [16] and with only three classified species of this genus known, reconstructing their full genomes and classifying novel ourmia-like viruses is particularly challenging. However, their presence here in such high quantities (11% of all detected viral contigs) suggests that they could potentially play an important, but as yet unknown role in grassland ecology. Although viruses are often thought of in terms of pathogenicity, some form persistent mutualistic relationships with their hosts [52] whilst others have been shown to trigger hypovirulent phenotypes in normally pathogenic plant fungi and are usable as biocontrol agents [53], creating the prospect that soil viruses may present opportunities for agricultural biotechnology applications.

*Pisuviricota* (Fig. 4b), was the most highly represented RNA virus phylum in this study, comprising 40% of identified viral contigs. This is a highly divergent group of viruses with a broad host range and so reliably identifying the specific host for individual viruses is challenging. Members of the family *Picornaviridae* infect vertebrates and often cause economically important infections [54]. Relatively few potential *Picornaviridae* were found in this study; however, those that were found occupied branches containing various bovine enteric viruses and could be derived from fertiliser manure or sheep dung. Although the separate fields of soil, plant and animal viromics are well established, there are few, if any, studies that consider these separate environments together in order to understand the flow of viruses between them at a community ecology scale.

Of particular interest here are the members of the family *Dicistroviridae* (Fig. 4b, bottom right, orange). These arthropod-infecting viruses can range from commensal to lethal disease-causing pathogens with significant economic consequences [55]. The dicistro-like viruses found in this study observable in the enlarged tree in Supplementary Fig. 8a divide into four clades: the reference viruses found within the first clade infect crustaceans and the two out of three viral contigs from this study were found in either two or all three coastal samples, the other found in one semi-improved site (Supplementary Fig. 8b). The other three clades contain insect-paralysis causing reference viruses, suggesting that soils may harbour arthropod viruses capable of acutely affecting local mesofauna populations. As soil mesofauna are critical to multiple soil functions [56], the diversity and ecological roles of arthropod and other invertebrate infecting viruses in soil ecosystems warrant further investigation.

In addition to +ssRNA viruses, viruses belonging to the bisegmented dsRNA *Partitiviridae* family are found within this group, and are capable of infecting plants, fungi and protozoa (see Supplementary fig. 9). Few viral contigs were found within this group, most likely because, as with *Narnaviridae*, members of the *Partitiviridae* are transmitted exclusively via intracellular mechanisms during spore formation in fungi or ovule/ pollen production in plants [57]. The *Partitiviridae* viruses found in this study may have been released from plant and fungal tissue in the soil during the extraction process or be present as free virions in the extracellular environment. Although this has not been demonstrated empirically here, it is possible to speculate that infection from normally obligate intracellular viruses could occur when mechanical damage occurs to plants and fungi in soils containing infectious and intact virions.

The viral contigs placed within the phylum *Kitrinoviricota* are distributed throughout the phylogenetic tree of known RNA viruses and are highly numerate, representing 32.4% of identified viral contigs (Fig. 4c). Of particular interest here are the three divergent clades in the bottom left quadrant, with the one on the far left containing many known members of the family *Tombusviridae* (blue). These viruses have a wide host range, including plants, protists, invertebrates and vertebrates. The nodaviruses (Fig. 4c, orange) divide into two categories: alpha-nodaviruses, predominantly isolated from insects but featuring a wide host range under laboratory conditions, and beta-nodaviruses, infecting fish [58]. The noda-like viral contigs identified in this dataset were relatively evenly distributed between the managed grassland and upland peat sites, but none were detected in the coastal grassland (Supplemental Fig. 10).

The phylum *Duplornaviricota* contains the majority of known dsRNA viruses and comparatively, few were detected. *Totiviridae* members (Fig. 4d—purple) infect fungi, protozoa, vertebrates and invertebrates [16]. Viral contigs identified in this study were predominantly found to cluster with isolates that infect animals (right hand side) but some could be found with the fungi-associated *Totiviridae* (left hand side). Very few viral contigs were found amongst possible *Reoviridae* (Fig. 4d—orange), with one contig (k127_2512471) showing 97% nucleotide sequence similarity to human rotavirus A (EF554115), found in samples coastal grassland-1 and semi-improved grassland-2.

Only four -ssRNA viral contigs were found in this study and poorly aligned with other known *Negarnaviricota*, which form a clade located within the Duplornaviricota (see Fig. 3a) [16, 19]. -ssRNA virus structure may inhibit detection by viromics: they are almost exclusively lipid-enveloped, occasionally lacking nucleocapsid proteins [59], and the harsh extraction protocol may lead to virion disruption and loss of viral RNA. This may also be due to the underrepresentation of plant and soil-dwelling arthropod *Negarnaviricota* within nucleotide databases hindering their detection. Examples of this clade are significantly biased towards vertebrate pathogens [60], however, increased use of high-throughput sequencing has rapidly expanded our breadth of knowledge of *Negarnaviricota* in plants [61].

## Conclusions

Using an altitudinal primary productivity gradient as a source of soils with contrasting ecological properties for RNA virome analysis, this study is the first to apply a direct viromics approach to examine the *in-situ* soil RNA viral community of soil ecosystems. We detected 3462 viral contigs across five sample sites, and observed site-specific variation in viral contig relative abundance. The viral contigs we detected are predicted to be from viruses of a range of hosts, including fungi, bacteria, vertebrates, invertebrates and plants. Therefore, RNA viruses have the potential to influence the grassland soil ecosystem at multiple trophic levels. From a technical standpoint, further development of both wet-lab and bioinformatics techniques is needed to further improve the detection and study of soil RNA viruses. Many RNA viruses have segmented and multipartite genomes, complicating the recovery of full RNA viral genomes from meta-transcriptomics and metaviromics datasets. This study found comparatively fewer putative mycoviruses compared to a previous study [12] examining RdRP containing contigs in soil meta-transcriptomics data. This may be due in part to the different structural characteristics and methods of dispersal used by viruses infecting fungi. While it has been shown that viromics outperforms metagenomics in the recovery of DNA viral genomes [22], the lack of capsid production in key clades of mycoviruses requires consideration when developing future soil RNA viral ecology methodologies. Use of paired metagenomics, meta-transcriptomics and DNA/ RNA viromics will potentially overcome this difference in detection between RNA viruses and further our understanding of how virus-host interactions and actively replicating viruses influence soil macro- and microbiology.

Whilst environmental DNA viromes are typically dominated by viruses of the prokaryote-infecting class *Caudoviricetes*, the balance in RNA viromes is heavily skewed towards eukaryotic viruses [62]. There are multiple explanations for this discrepancy. Many current DNA virus discovery tools are tuned to detect prokaryotic viruses and so may not detect distantly related eukaryotic viruses, however, BLAST-based studies report similar biases towards prokaryotic DNA viruses [63, 64]. Reference databases of RNA viral sequences are also biased towards viruses of eukaryotes [20] and so HMM-based search strategies may be more sensitive to these clades due to biases in the underlying HMMs they are based on. These discrepancies could also be due to evolutionary bottlenecks creating a genuine difference in the number of viruses of each domain of cellular life found in terrestrial and aquatic environments. The development of specialist tools for detecting novel eukaryotic DNA viruses and/ or prokaryotic RNA viruses and further exploration of the RNA viral communities of different ecosystems will aid in assessing the true extent of the overall RNA virosphere.

The impact that soilborne RNA viruses have on their host organisms has only just started to be explored, and future work is needed to establish the many influences they may have on global terrestrial ecosystems. Grassland soil bacterial communities show clear responses to the effects of climate change that are mediated by plant–soil–microbial interactions [65] and viruses have the potential to influence soil nutrient cycling through host metabolic reprogramming [6] and their effects on soil microbial community dynamics [12] similarly to marine viral communities [66]. Our work demonstrates that RNA viral communities are heavily influenced by location, with upland peatland and unmanaged coastal grassland soils sharing very few viral contigs with managed grassland ecosystems and also showing broad differences at the phylum level. Soilborne RNA viruses identified in this study potentially infect hosts across a wide range of trophic levels and can therefore influence soil ecosystems at a variety of scales. Linking these effects of soilborne RNA virus-host interactions with naturally occurring and anthropogenic environmental processes, will be critical in developing a complete picture of how soil ecosystems respond to environmental change.

## Supplementary information

Supplementary Information

## Data Availability

Post-sequencing centre QC reads are available from the European Nucleotide Archive (BioProject accession number PRJEB45714). Assembled viral contigs were deposited at DDBJ/ENA/GenBank under the accession JAKNTR000000000. The version described in this paper is version JAKNTR010000000. The parent BioProject accession number for all sequencing data is PRJNA804556. RdRP protein sequences, custom R scripts used for data analysis, required input files, multiple sequence alignments and phylogenetic trees are available from Github (https://github.com/LSHillary/RnaSoilVirome).
