# Supplementary material for: RNA-viromics reveals diverse communities of soil RNA viruses with the potential to affect grassland ecosystems across multiple trophic levels
Source: ISME Commun. 2022 Apr 8;2:34. doi: 10.1038/s43705-022-00110-x (PMC8992426; doi:10.1038/s43705-022-00110-x)
Supplement: Supplementary file 1 — Supplementary Information [file 43705_2022_110_MOESM1_ESM.pdf]

RNA-viromics reveals diverse communities of soil RNA viruses with the potential to affect grassland ecosystems across multiple trophic levels

Luke S. Hillary, Evelien M. Adriaenssens, David L. Jones and James E. McDonald

Supplementary Table 1 – Sampling site descriptions.

	Site	Location	Soil classification	Soil description	Altitude (m asl)	pH	Electrical conductivity (µS cm^{-1})	Total carbon (%)
A	Upland peatland	53° 13' 1.22" N 4° 1' 8.78" W	Non-calcaric lithosol	Very acid upland soil with a wet highly organic topsoil	431	4.27	39	29.1
B	Upland grassland	53°13'33.00" N 4° 0' 54.86" W	Typical podzolic brown soil	Freely draining acid loamy sandy clay soil over rock	289	5.89	30	11.3
C	Semi-improved grassland	53° 13' 55.24" N 4° 1' 2.22" W	Typical podzolic brown soil	Freely draining slightly acid sandy loam soil	77	4.61	27	11.2
D	Lowland grassland	53° 14' 10.98" N 4° 1' 1.74" W	Typical orthic brown soil	Sandy clay loam, freely draining sheep-grazed soil	19	5.78	42	3.62
E	Coastal grassland	53° 14' 34.17" N 4° 1' 18.78" W	Saline alluvial gley soil	Silt-textured, poorly draining soil with periodic tidal inundation	3	8.03	1810	2.89

Soils were classified according to Avery (1990). The major properties of the sites and soils are shown in Table S1 above, while a general description of the catena sequence is provided in Farrell et al. (2014), Shaw et al (2014) and Withers et al. (2020). The altitudinal gradient represents a primary productivity gradient with more intensive agricultural production at Site D which receives regular fertiliser (N, P and K) and lime applications. The mean annual temperature at the bottom and top sites was 10.2 and 7.3 °C respectively, while the gradient in annual rainfall was 1065 to 1690 mm, respectively. All sites had a different vegetation cover (all dominated by grasses) and were grazed by Welsh mountain sheep (*Ovis aries* L.). Soil pH and electrical conductivity were measured in 1:2.5 (w/v) soil-to-distilled water extracts using standard electrodes. Total C and N were determined on a TruSpec CN analyser (Leco Corp., St Joseph, MI). Site E is a soil developed on recent marine deposits and

contains $CaCO_3$ from shell deposits. Its pedogenic age is ca. 500 years. All other sites have a pedogenic age of ca. 10,000 years. Site A is developed on a rhyolite parent material, Sites B and C on Ordovician age schist and shale, and Site D on mixed glacial till (rhyolite, mudstone, slate, shale, microdiorite). Sites D and E rarely undergo freezing, while Sites A-C experience periodic freezing in winter with winter snow cover often present at Site A. The vegetation at Site A comprises *Festuca ovina* L., *Juncus effusus* L. and *Trichophorum cespitosum* (L.) Hartman. The vegetation at Site B is dominated by *Agrostis canina* L., *Agrostis capillaris* L., *Anthoxanthum odoratum* L. and *Potentilla erecta* (L.) Rauschel. The vegetation at Site C is dominated by *Festuca ovina* L. and *Pteridium aquilinum* L.. Site D is dominated by *Lolium perenne* L. and *Trifolium repens* L. while the vegetation at site E is dominated by *Plantago maritima*, *Festuca* sp. and *Salicornia europaea.* Grazing intensity decreases with altitude due to the decline in primary productivity. All soils are free draining, with the exception of Site E which periodically experiences coastal inundation at spring tides (i.e. leading to the high EC values observed in Table 1) and has anaerobic features (Fe^{3+}/Fe^{2+} mottles and FeS production). The texture of the mineral soils is as follows: Sites B to D, sandy clay loam, Site E silty sand. The humification status of the peat at Site A is H5 on the von Post scale indicating a moderate degree of humification (Okono et al., 1981). Sites B and D have earthworms present, although these are much more abundant at Site D where intensive bioturbation has led to the development of a crumb structure and Eutric Cambisol horizon structure (Ah, Bw, C). The structure at Site C is described as granular while at Site E it is classified as massive, lacking macropores. The bulk density of the soils ranged from 0.45 g cm^{-3} at Site A to 1.15 g cm^{-3} at Site E with no site showing signs of compaction that would inhibit root growth.

References

Avery, B.W. (1990) Soils of the British Isles. CAB International, Wallingford, UK.

Ekono (1981) Report on energy use of peat. Ekono Inc., Report of the United Nations Conference on New and Renewable Sources of Energy, Nairobi, 10 to 21 August 1981, United Nations. New York.

Farrell, M., Macdonald, L.M., Hill, P.W., Wanniarachchi, S.D., Farrar, J., Bardgett, R.D., Jones, D.L. (2014) Amino acid dynamics across a grassland altitudinal gradient. Soil Biology and Biochemistry 76, 179-182.

Shaw, R., Williams, A.P., Jones, D.L. (2014) Assessing soil nitrogen availability using microdialysis-derived diffusive flux measurements. Soil Science Society of America Journal 78, 1797-1803.

Withers, E., Hill, P.W., Chadwick, D.R., Jones, D.L. (2020) Use of untargeted metabolomics for assessing soil quality and microbial function. Soil Biology and Biochemistry 143, 107758.

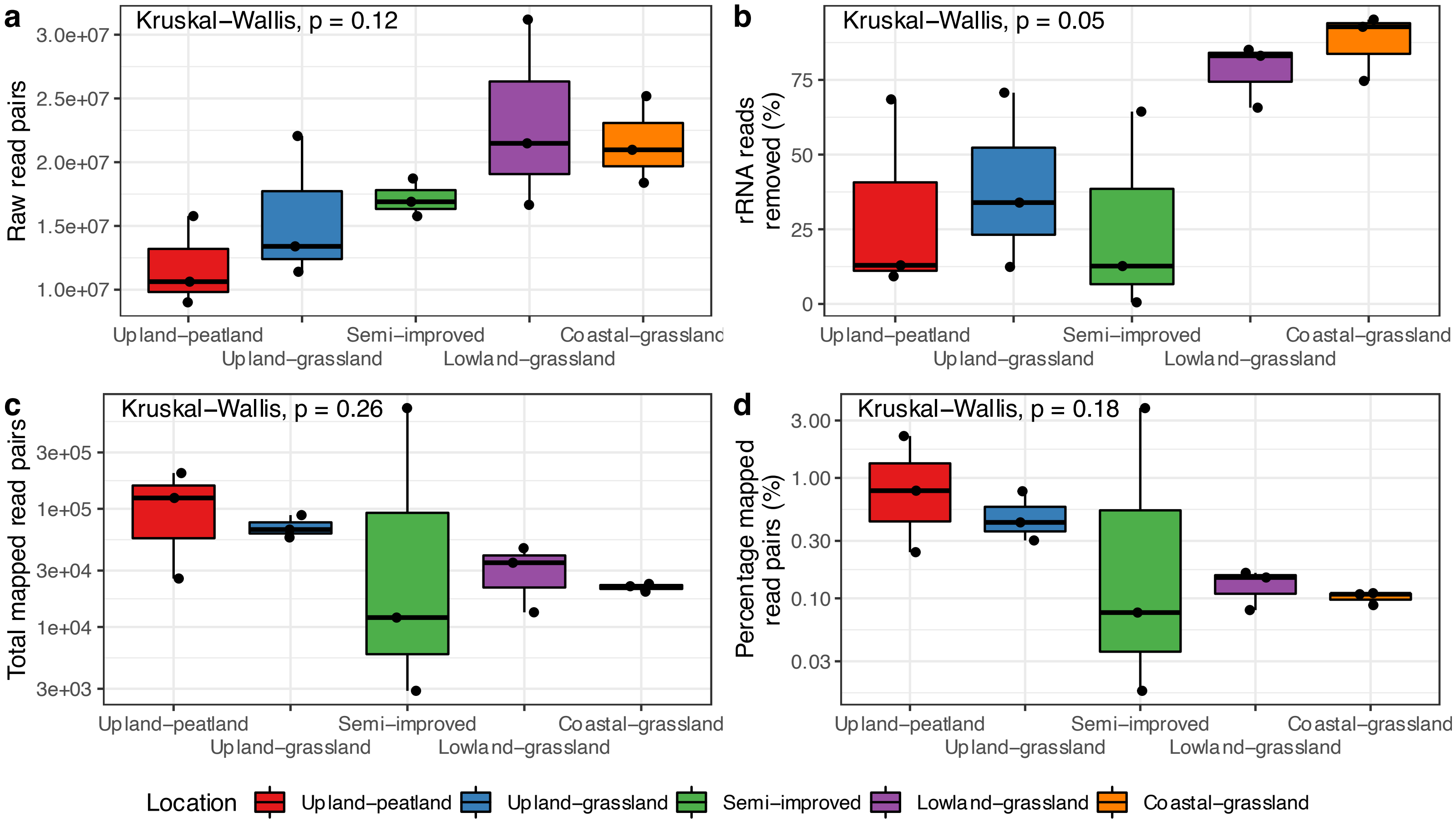

Supplementary Figure 1 – Total raw reads (a), percentage of rRNA reads (b), and total and percentage (d) read pairs mapped to viral contigs at each location. No significant overall effect from location was observed on these metrics (Kruskal-Wallis) and no significant pairwise interactions were observed (all adjusted p-values >0.2). No correlations were found between raw read pairs and rRNA reads removed or

between rRNA reads removed and the percentage of reads mapping to viral contigs (Spearman rank correlation, p = 0.119 and p = 0.611 respectively).

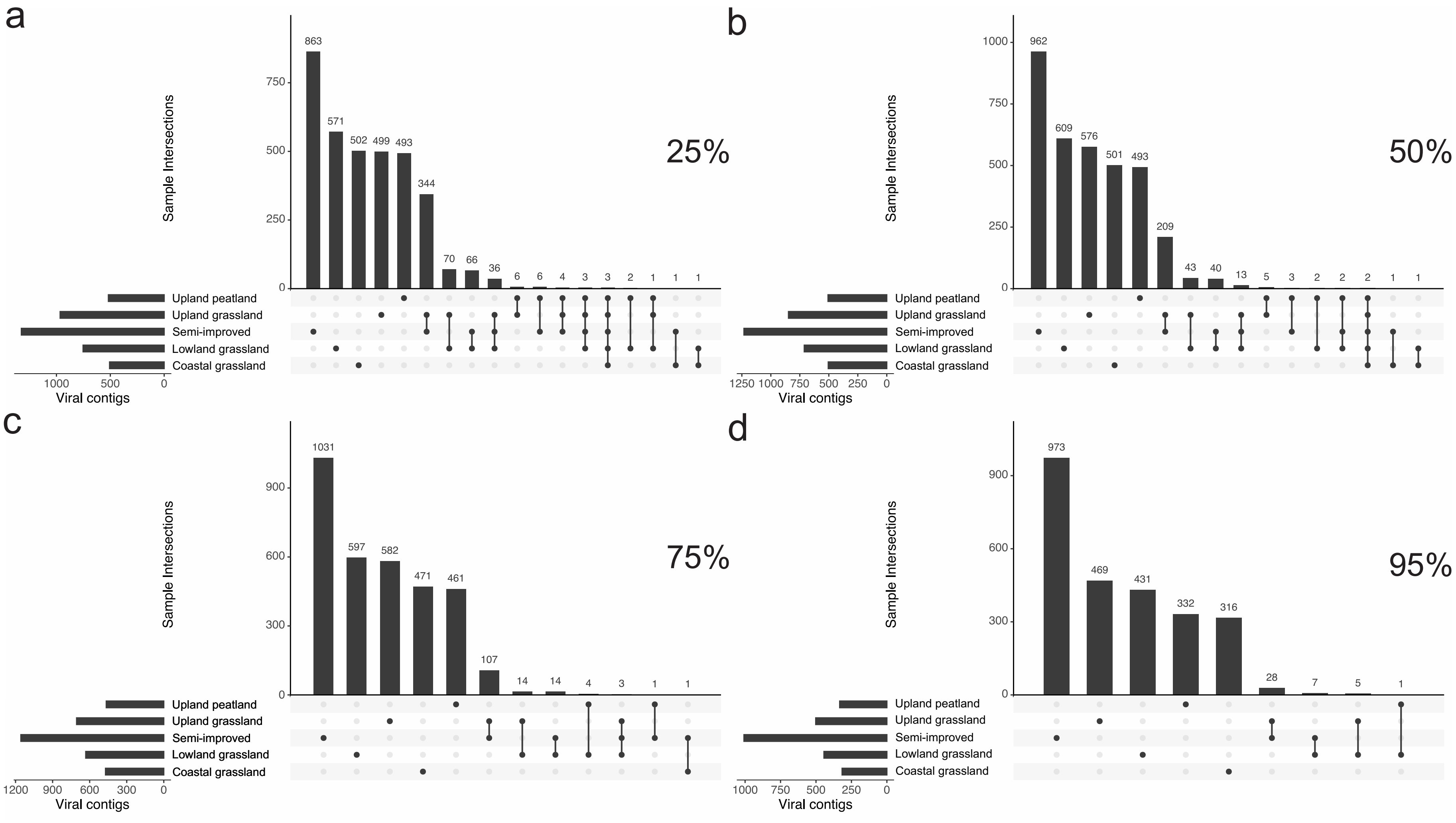

Supplementary Figure 2 – Comparison of co-occurring vOTUs at (a) 25%, (b) 50%, (c) 75% and (d) 95% horizontal genome coverage thresholds. Similar conclusions on vOTU sharing between sampling sites when varying the alignment fraction required to judge a vOTU present within a sample. vOTUs are most commonly found within one site with the grassland sites sharing more common vOTUs than other habitats. The costal grassland site consistently shared the least number of vOTUs with the other habitats.

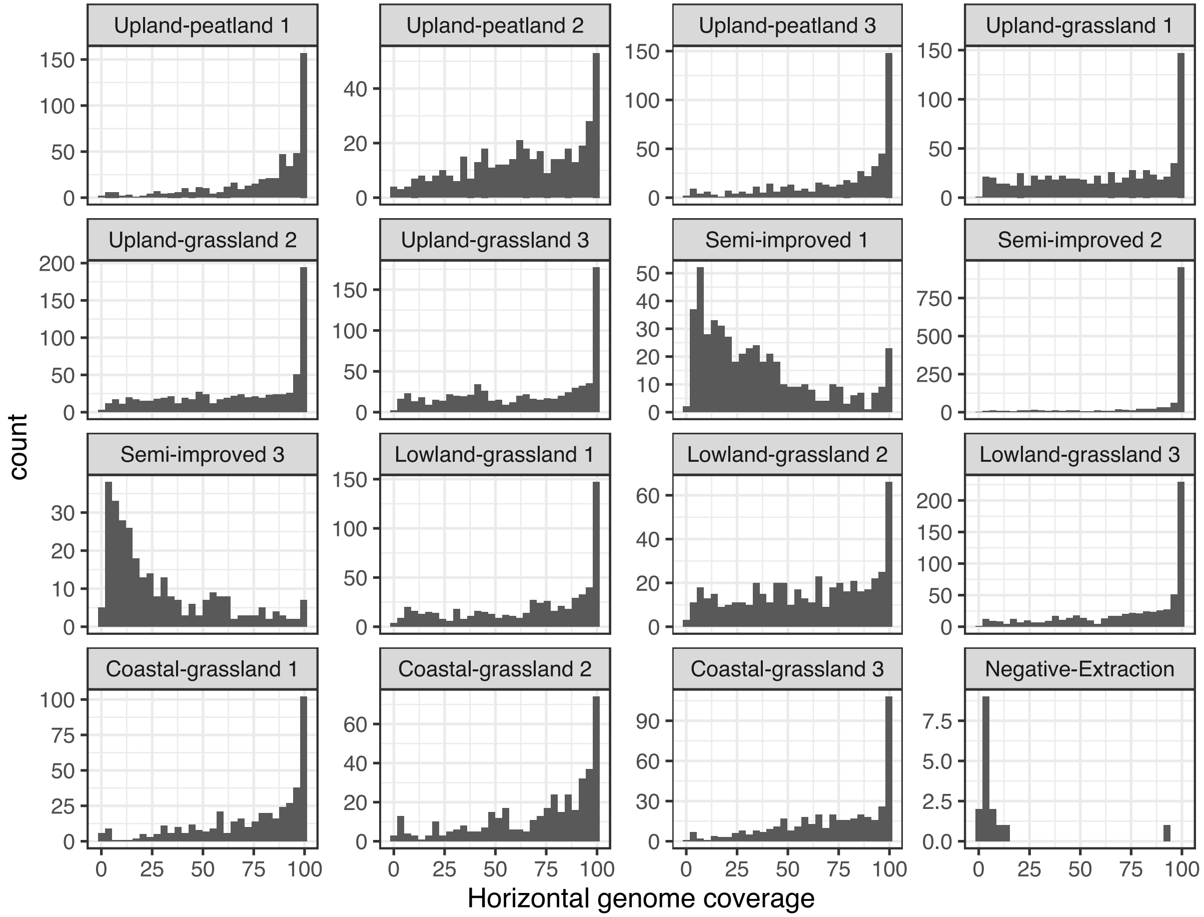

Supplementary Figure 3 - Histograms of horizontal genome coverage for all contigs where coverage was >0%. Some samples show the majority of viruses with coverages close to 100% (e.g. Upland grassland samples), whilst others show a range of values. Other samples show an increase in the number of genomes from 50%-100%, whilst remaining relatively flat, or decreasing from 0-50% (e.g. Semi-improved 1). A horizontal genome coverage threshold of 50% was chosen as a compromise between preventing false-positives from short contigs being covered by one read and false negatives from longer viral contigs that may be present at low abundances in some samples.

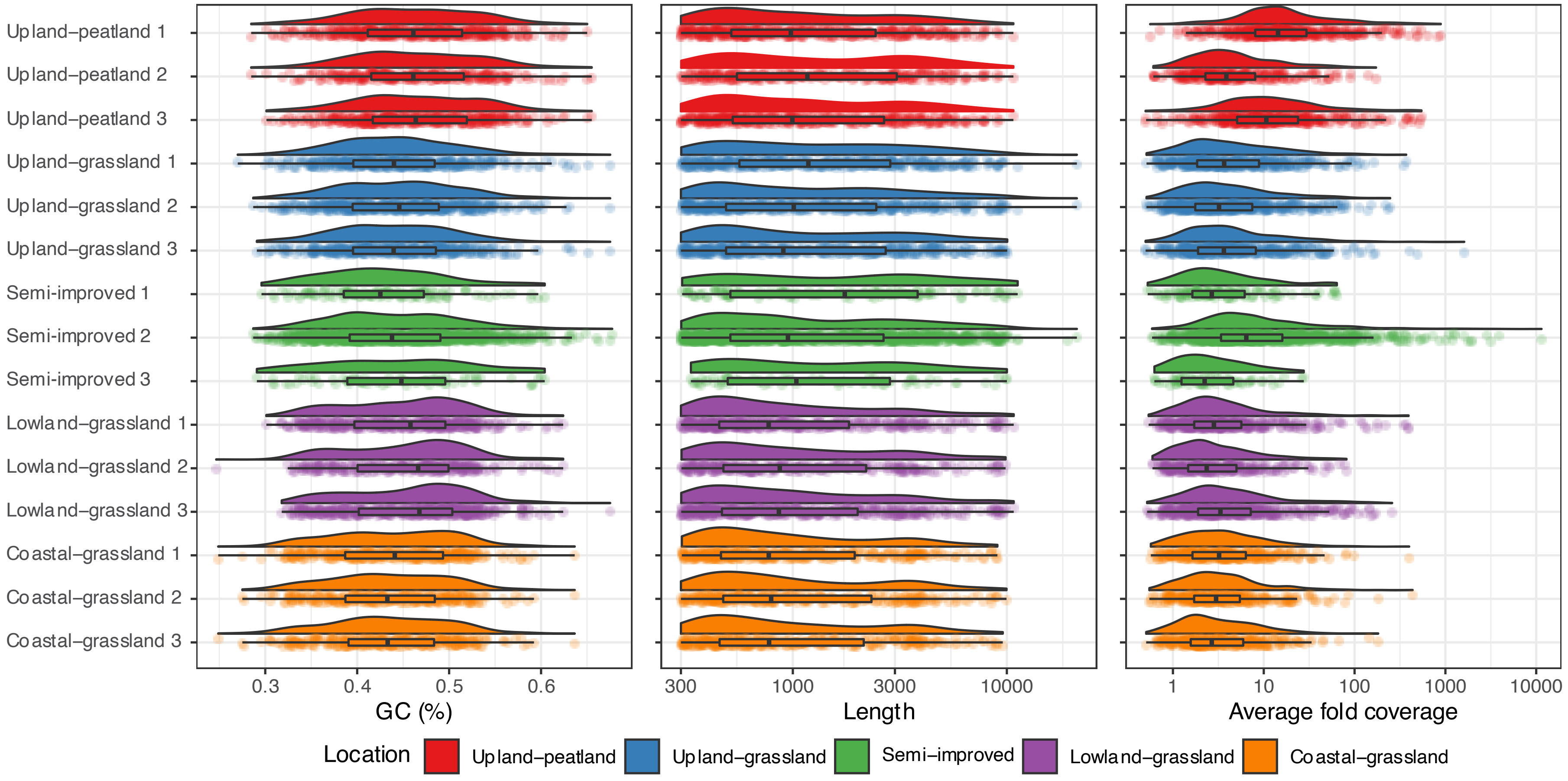

Supplementary Figure 4 – GC content, length and vertical average fold coverage of viral contigs with >50% horizontal coverage within each sample. Note that plots for length and average fold coverage are displayed on a log scale.

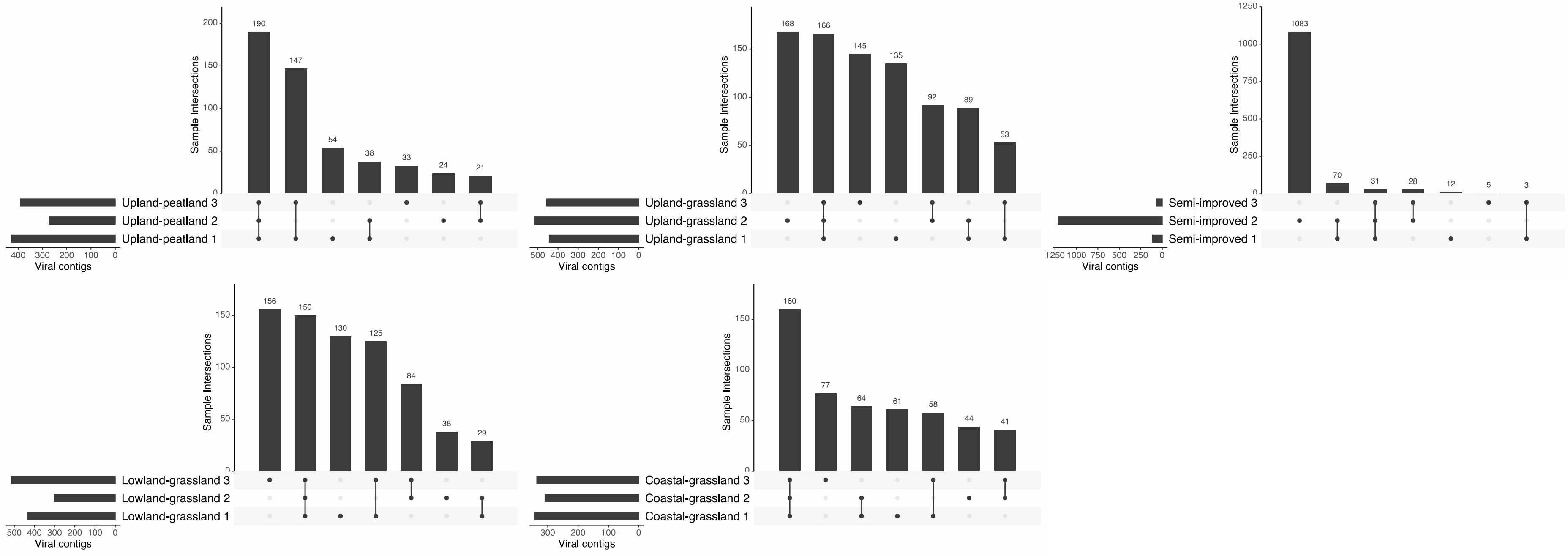

Supplementary Figure 5 – UpSet plots of viral contigs shared between sampling replicates. The intersection between all three replicates is the largest, or second largest at all sampling sites except for the semi-improved site, where the majority of viral contigs were identified in replicate 2. Although replicate 2 had fewer post-rRNA read removal, more reads aligned to viral contigs suggesting local heterogeneity in the quantity of VLPs at this site. All other samples had comparable numbers of viral contigs per sample.

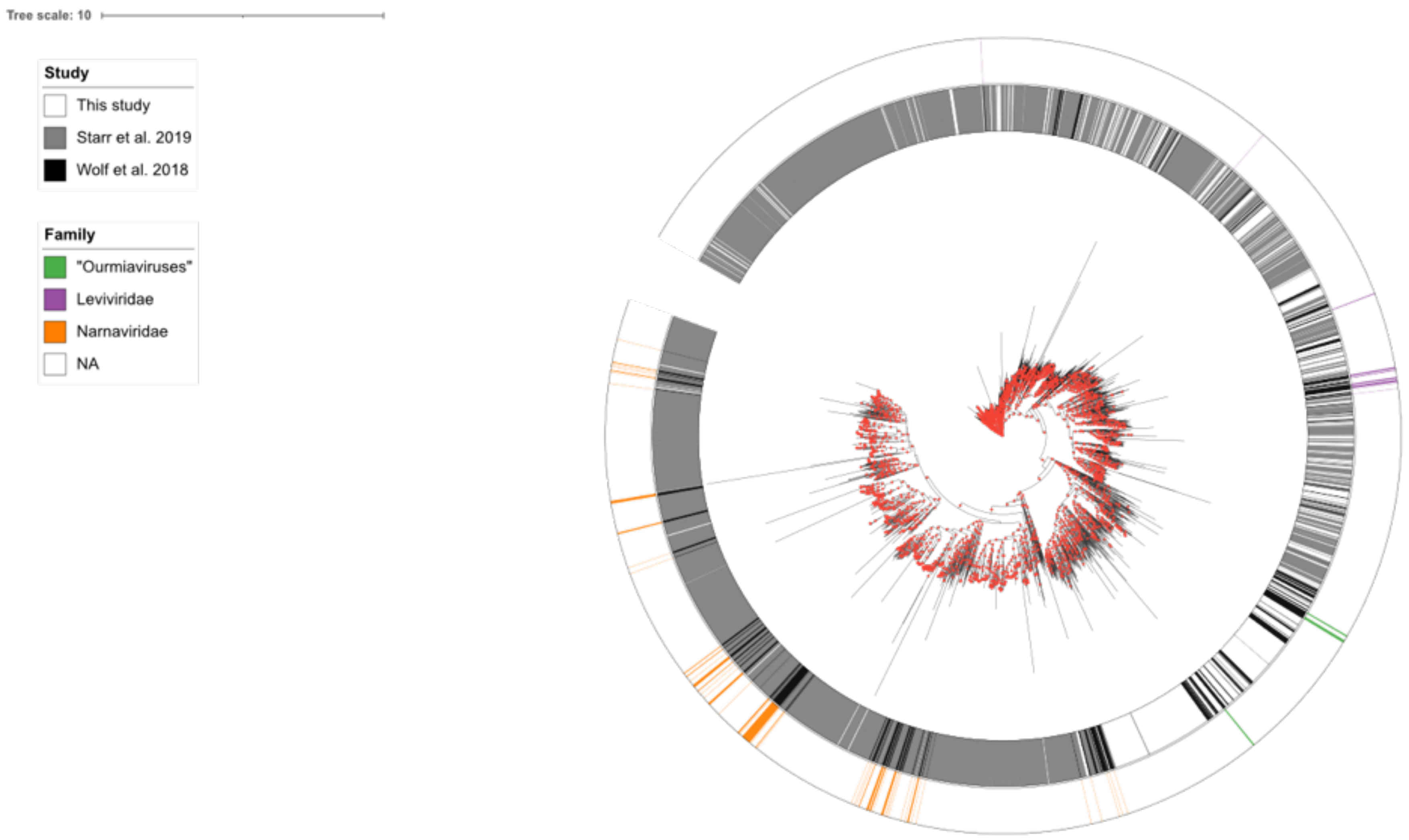

(a) Lenarviricota

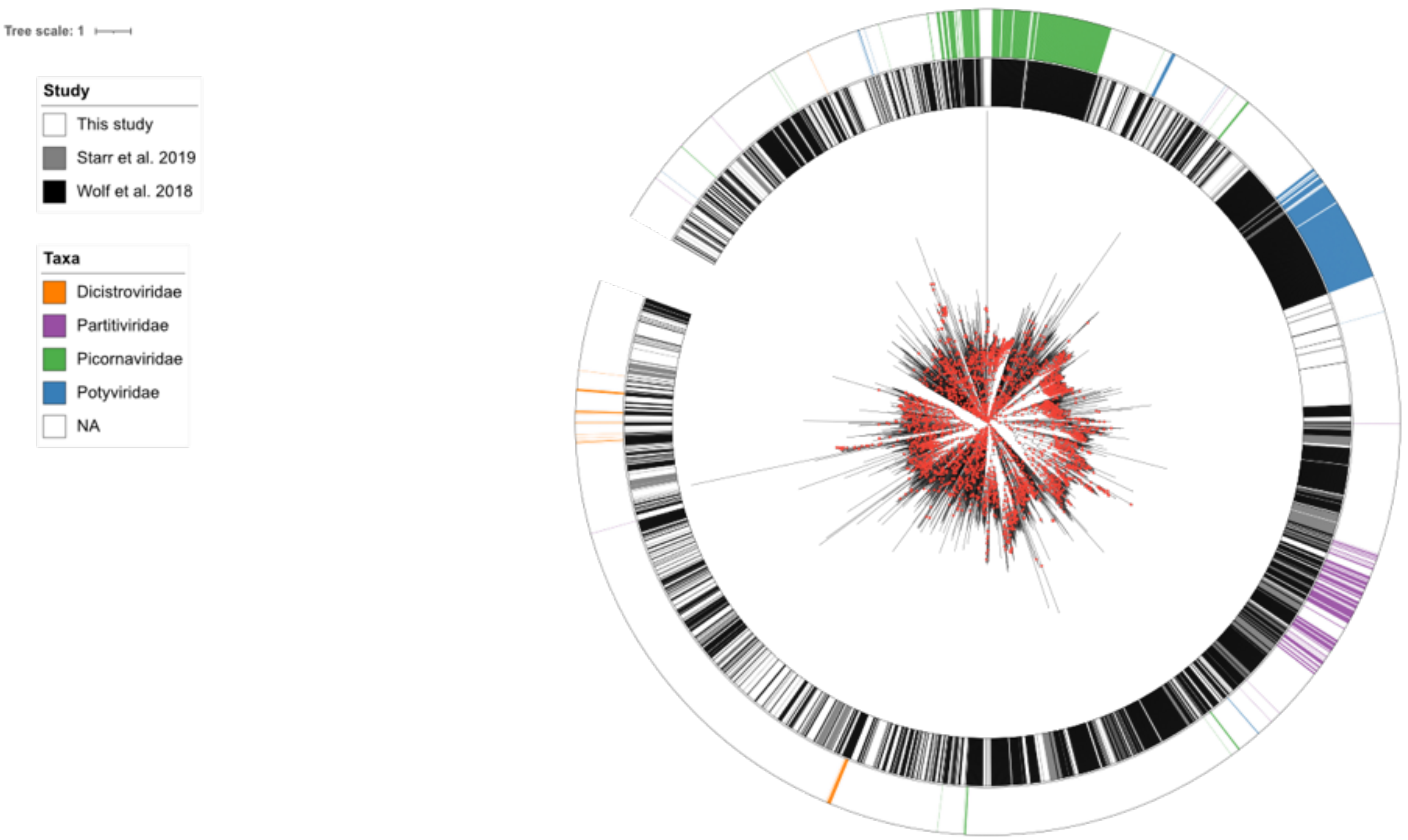

(b) Pisuviricota

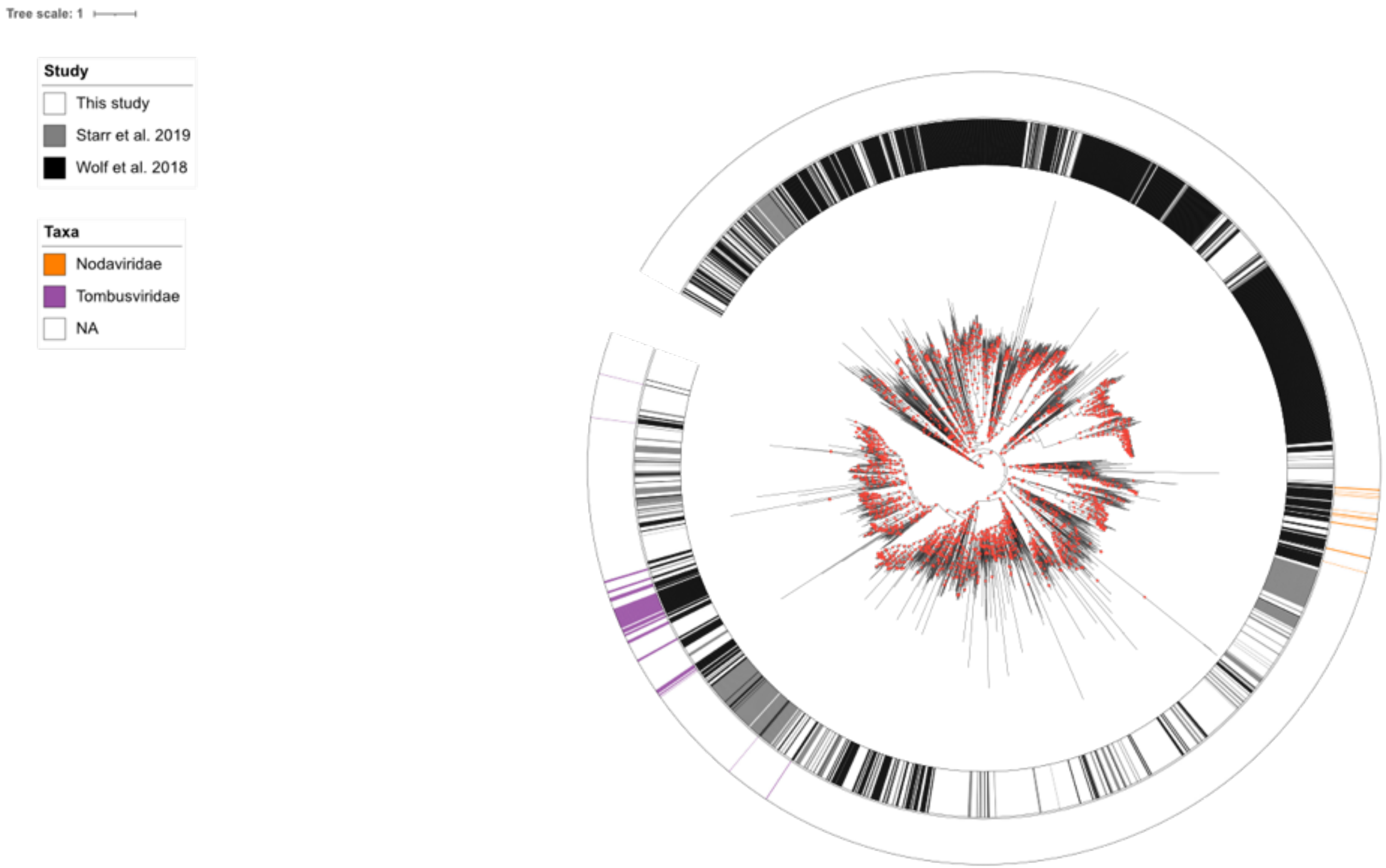

(c) Kitrinoviricota

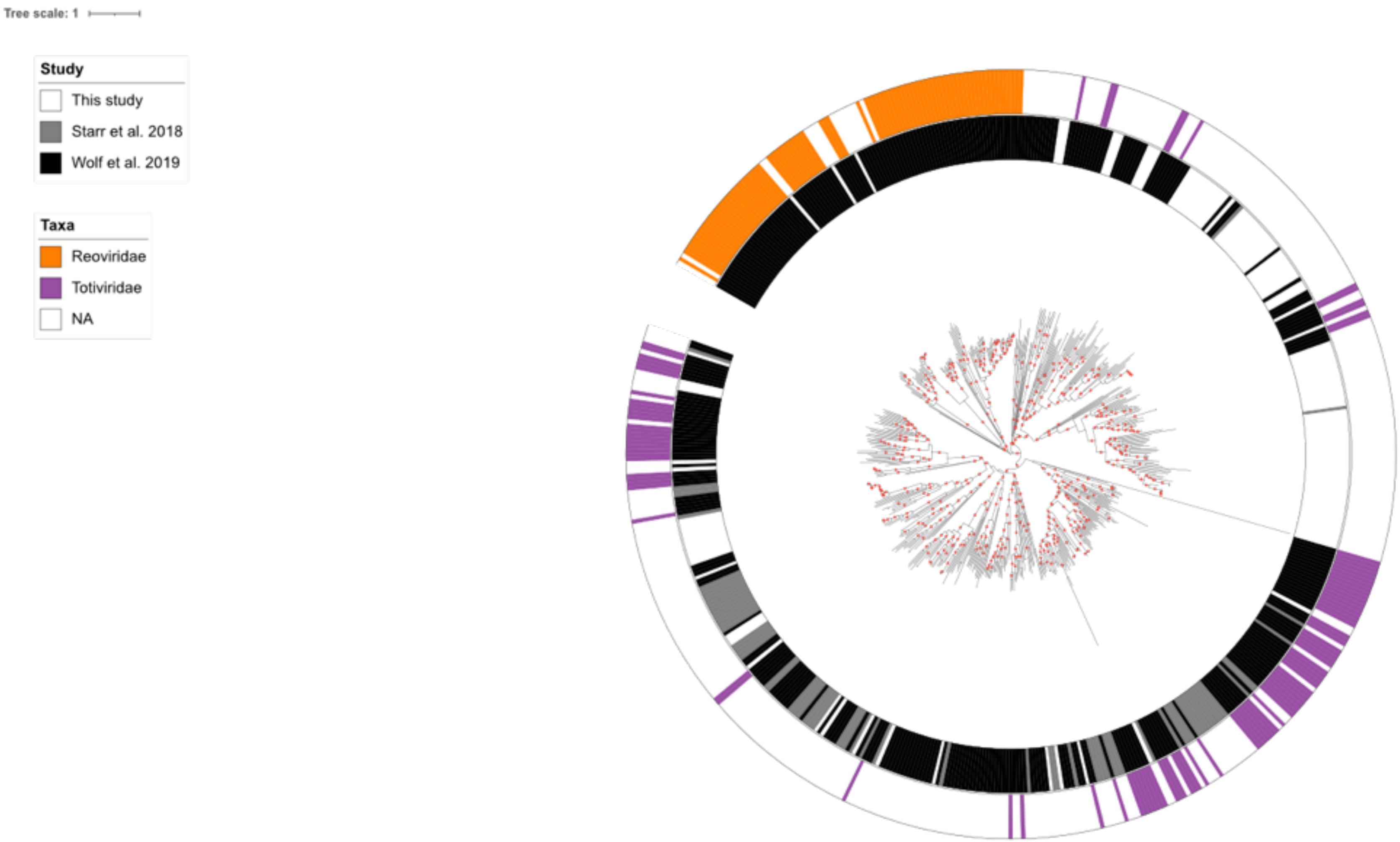

(d) Duplornaviricota

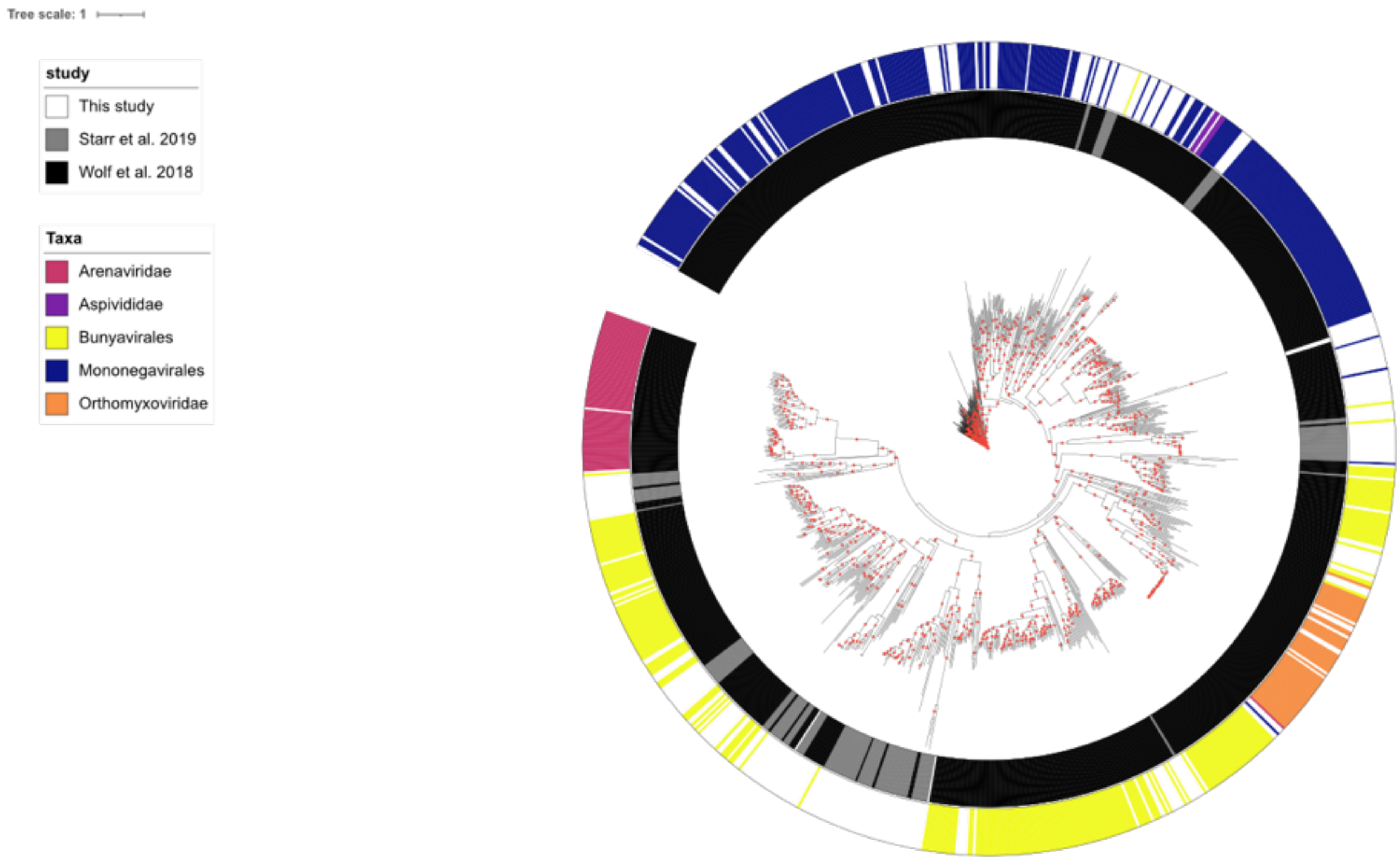

(e) Negarnaviricota

Supplementary Figure 6 (previous pages) – enlarged phylogenetic trees corresponding to those shown in figure 4 in the main mansucript. Branches with branch support ≥0.6 are indicated by red circles.

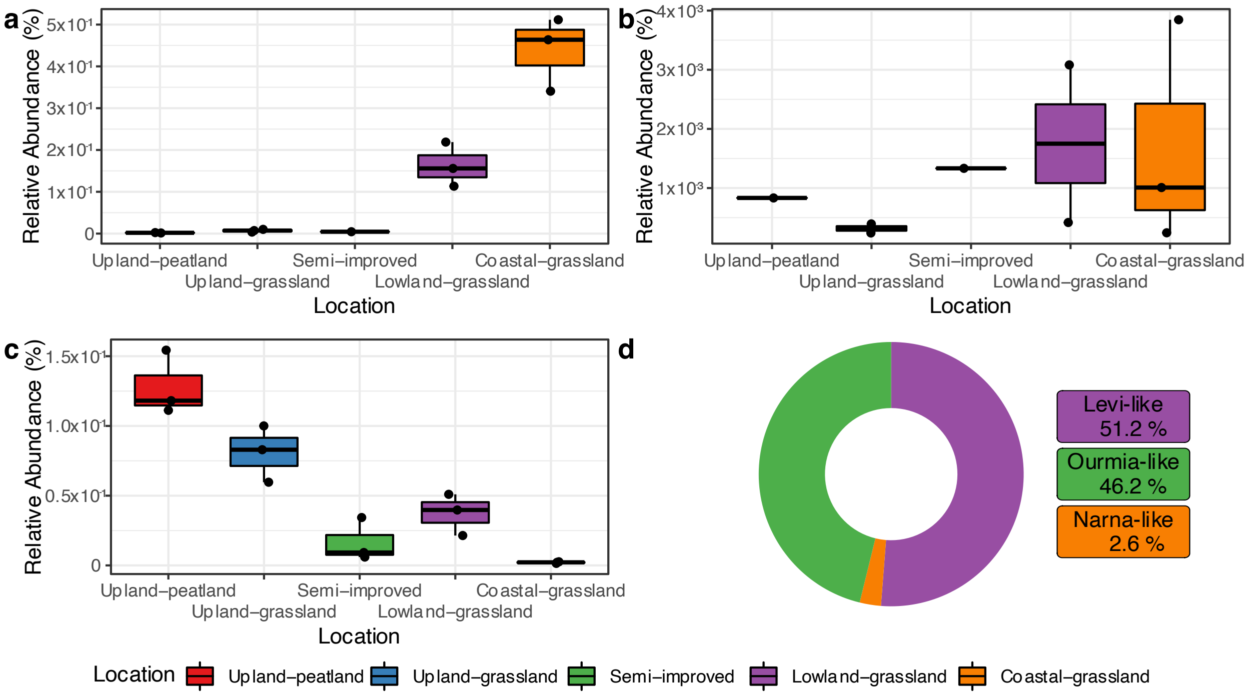

Supplementary Figure 7 – Boxplots of relative abundance of Levi-like (a), Narna-like (b) and Ourmia-like (c) viruses within the five soil-types sampled in this study. Location had a significant effect on the relative abundance of Levi-like and Ourmia-like viruses but not on Narna-like viruses (Kruskall-Wallis, X^2 = 10.3, 3.38, 13.2 and p = 0.0357, 0.497, 0.0102 respectively). (d) Doughnut plot of percentages of viral contigs from this study identified as Levi-like, Ourmia-like and Narna-like viruses.

(a)

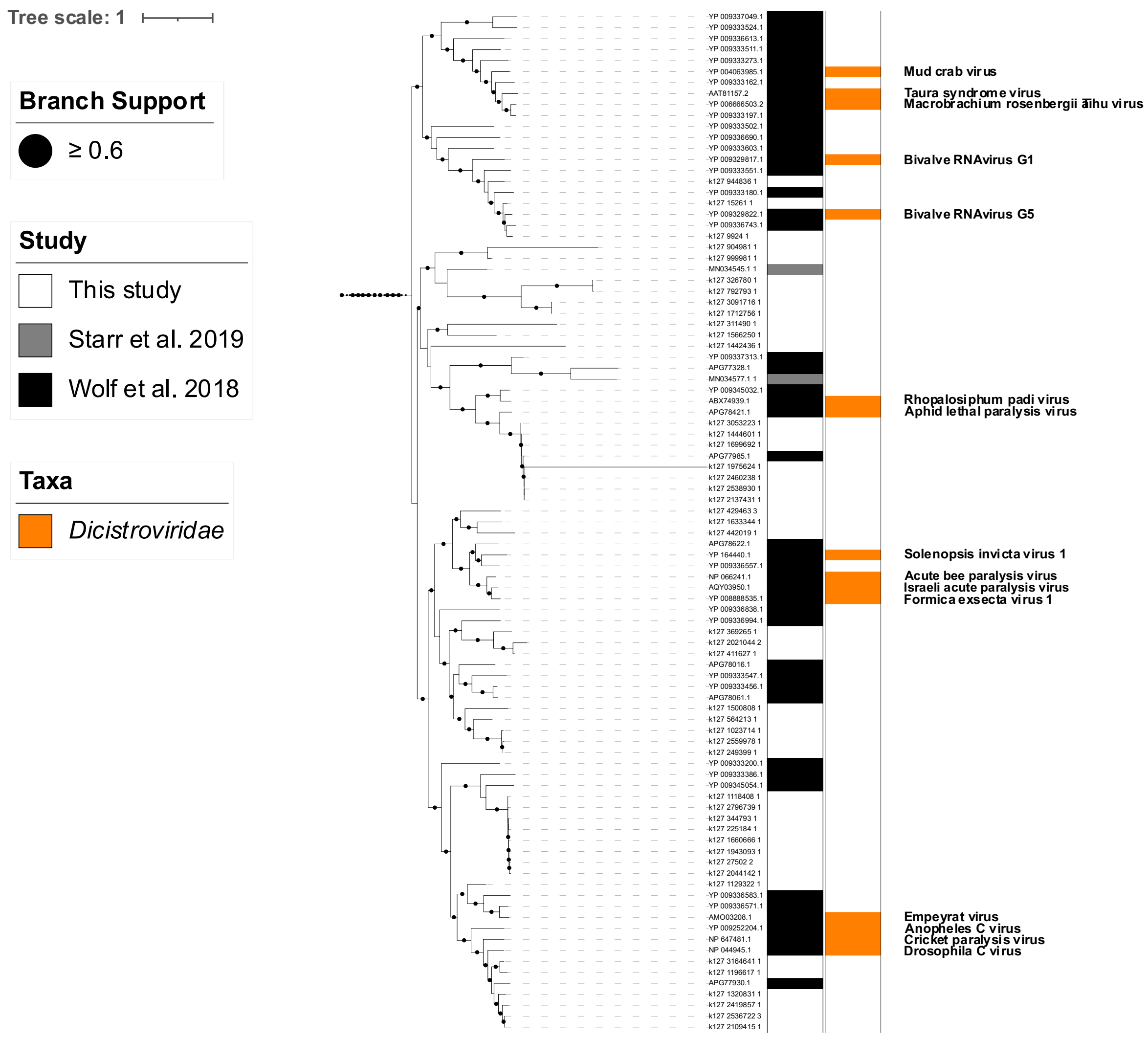
Tree scale: 1
Branch Support
≥ 0.6
Study
This study
Starr et al. 2019
Wolf et al. 2018
Taxa
Dicistroviridae
Mud crab virus
Taura syndrome virus
Macrobrachium rosenbergii Taihu virus
Bivalve RNAvirus G1
Bivalve RNAvirus G5
Rhopalosiphum padi virus
Aphid lethal paralysis virus
Solenopsis invicta virus 1
Acute bee paralysis virus
Israeli acute paralysis virus
Formica exsecta virus 1
Empeyrat virus
Anopheles C virus
Cricket paralysis virus
Drosophila C virus

(b)

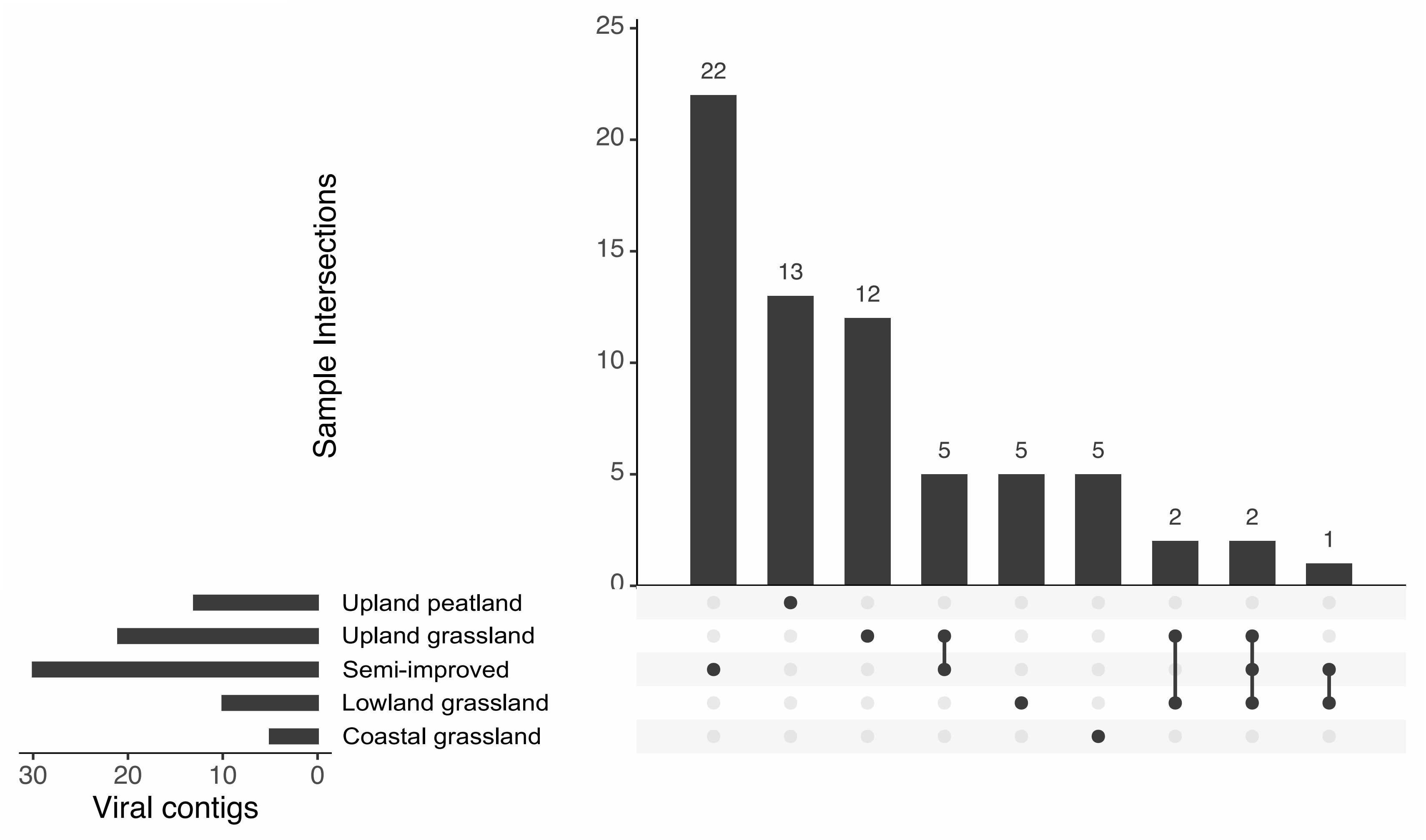

Supplementary Figure 8 – Pruned phylogenetic tree (a) and site distribution (b) of putative dicistro-like viruses. Viruses were predominantly found within upland/ semi-improved sites.

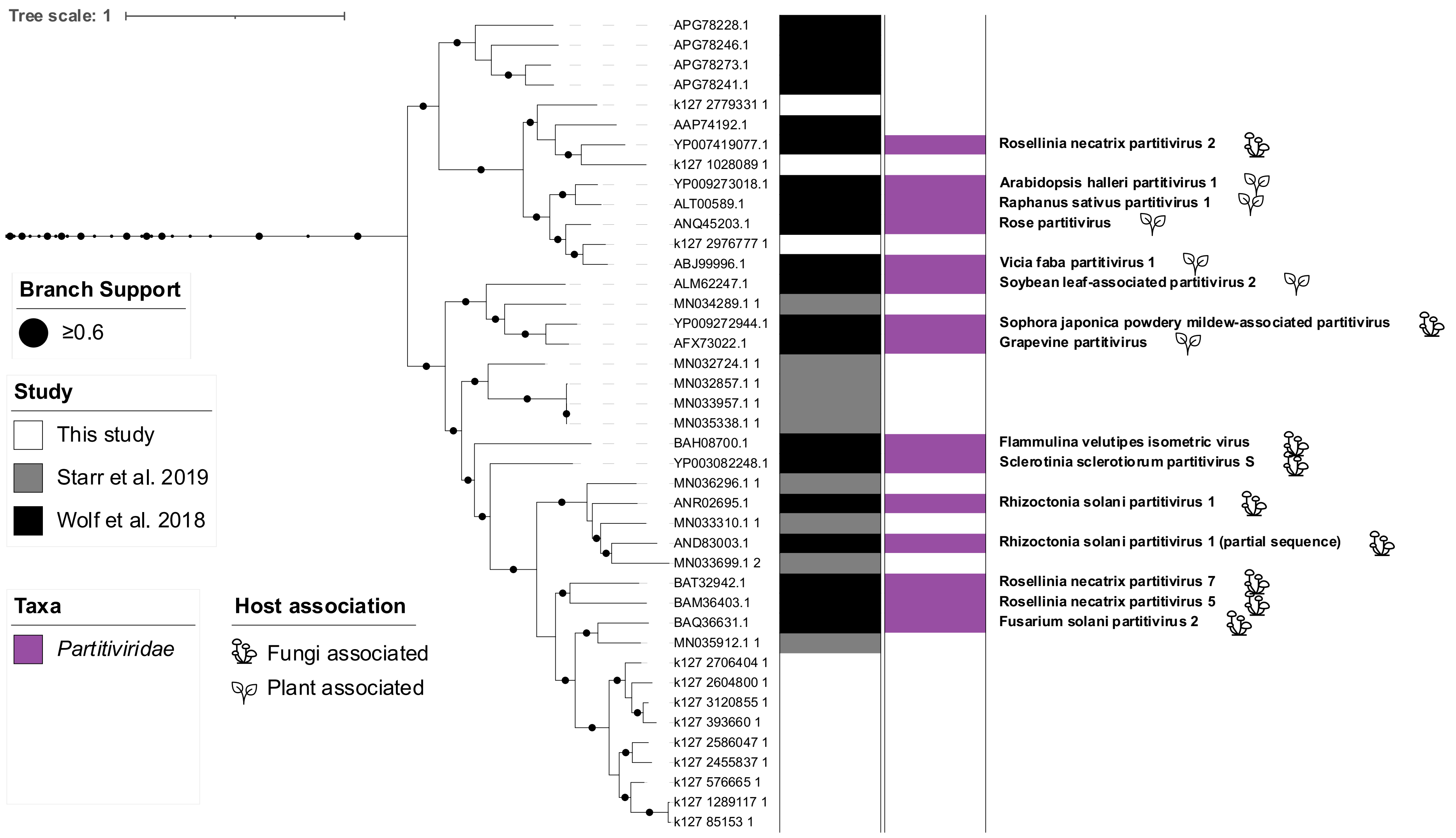

Supplementary Figure 9 – Pruned phylogenetic tree of putative partiti-like viruses. The majority of partiti-like vOTUs identified in this study are relatively closely related to Fusarium solani partitivirus 2 (indicated by short branch lengths at the bottom of the figure).

(a)

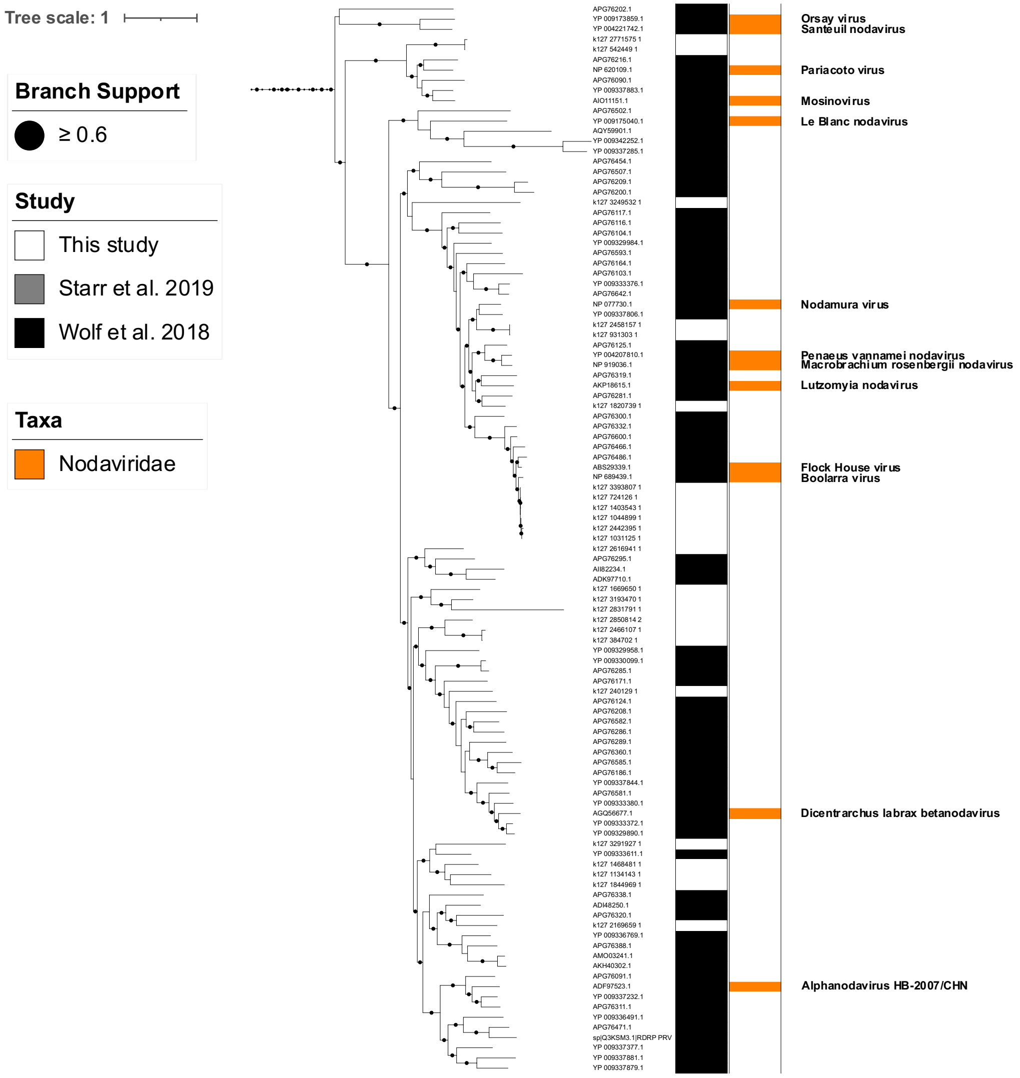
Tree scale: 1
Branch Support
≥ 0.6
Study
This study
Starr et al. 2019
Wolf et al. 2018
Taxa
Nodaviridae
Orsay virus
Santeuil nodavirus
Pariacoto virus
Mosinovirus
Le Blanc nodavirus
Nodamura virus
Penaeus vannamei nodavirus
Macrobrachium rosenbergii nodavirus
Lutzomyia nodavirus
Flock House virus
Boolarra virus
Dicentrarchus labrax betanodavirus
Alphanodavirus HB-2007/CHN

(b)

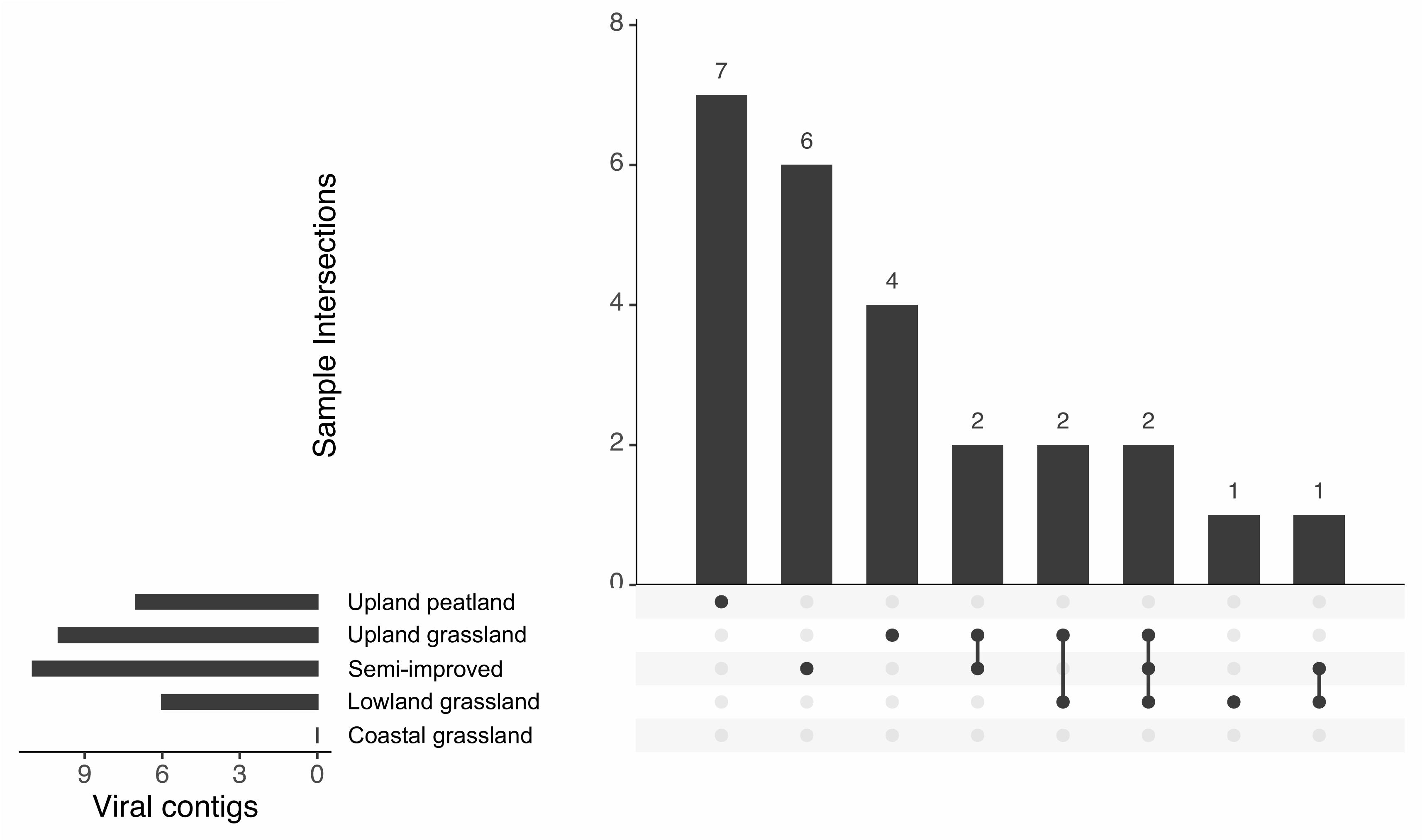

Supplementary Figure 10 – Enlarged phylogenetic tree (a) and site distribution (b) of noda-like viruses found in this study clustering near reference *Nodaviridae* RdRP sequences (a). Viral sequences were predominantly found in upland areas, with no noda-like viruses found in the coastal grassland site.
